# Population structure and genetic diversity of a coffee germplasm collection in China revealed by RAD-seq

**DOI:** 10.3389/fpls.2025.1629553

**Published:** 2025-09-04

**Authors:** Xinlei Jiang, Cheng Liu, Guanrun Ma, Mingzhu Zhao, Meifang Li, Tianming Chen, Pingxiang Zhao, Jingmin Wang, Qin Luo, Tieying Guo, Linlin Su, Zhirun Zhang, Jiayi Wang, Ziwei Xiao, Bing Xiao, Hua Zhou, Jinhong Li, Xuehui Bai

**Affiliations:** Yunnan Dehong Institute of Tropical Agricultural Science, Ruili, China

**Keywords:** *Coffea*, germplasm collection, RAD-seq, SNP markers, genetic diversity analysis, population structure analysis, sustainable agriculture

## Abstract

Coffee (*Coffea* spp.), a globally important crop, faces challenges in germplasm conservation due to habitat loss, climate change, and limited genetic diversity validation. This study aimed to evaluate the genetic representativeness of a coffee germplasm collection (CCGC, n=185) spanning major global varieties and wild relatives using re-striction-site associated DNA sequencing (RAD-seq). We performed genome-wide SNP profiling (37,729 loci), population structure analysis (STRUCTURE, PCA), and selection sweep detection (π) to assess genetic diversity, differentiation, and functional gene coverage. Results demonstrated that CCGC captured 98% of known disease-resistance loci (e.g., *SH3*, *RppM*) and exhibited high genetic diversity (π=0.1456, He=0.3014). Population structure analysis (K=3) identified three genetically distinct subgroups, among which Group 2 exhibited the highest diversity (He=0.3014, comparable to global coffee genetic resources) and encompassed all known *Hemileia vastatrix* resistance loci. The SNP density (7.5× higher than 5K SNP arrays) enabled precise identification of 47 selective sweep regions linked to domestication and adaptation. These findings validate CCGC as a genomically representative resource for coffee breeding and conservation. This work advances coffee genetic research by bridging resource preservation with molecular breeding strategies to address climate resilience and sustainable production.

## 1 Introduction

Coffee (*Coffea* spp.) is a perennial evergreen shrub or small tree in the *Rubiaceae* family, classified under the *genus Coffea* (Davis et al., 2011). The genus comprises approximately 124 species, with global coffee germplasm resources predominantly concentrated within the “coffee belt” (25°N to 25°S) (Davis et al., 2021). *Coffea arabica* an allotetraploid species (2n=4x=44 chromosomes), originated from hybridization between the diploid *Coffea canephora* and *Coffea eugenioides* (Anthony et al., 2002; Pagani et al., 2012; Cenci et al., 2012; Clarindo and Carvalho, 2008; Lashermes et al., 1999; Merot-L’anthoene et al., 2019). According to their genetic background and breeding methods, *Coffea arabica* varieties can be divided into three categories: *Bourbon*/*Typica* group, Ethiopian native group and gene infiltration group. The *Bourbon*/*Typica* group consists mainly of the traditional varieties *Bourbon*, *Typica* and their derivatives such as *Caturra*, which are known for their superior flavor but are less resistant to disease (Anthony et al., 2001). The Ethiopian native group is derived from local germplasm resources in Ethiopia, such as *Geisha*, and has unique flavor characteristics and rich genetic diversity (Zeru et al., 2012; Scalabrin et al., 2020). Introgression groups are disease-resistant varieties selected by interspecific hybridization, typically including the *Catimor* (*Caturra* × *Timor*) and *Sarchimor* (*Villa Sarchi* × HDT) series (Bertrand et al., 2005; Prakash et al., 2005). As a globally consumed beverage, coffee holds significant economic and cultural importance, serving as a vital pillar of agricultural economies and international trade for many developing countries (Davis et al., 2012). From Italian espresso to traditional Turkish brewing methods, coffee culture is deeply rooted around the world. Today, coffee culture has formed an important part of social interaction, artistic expression and lifestyle (Errington et al., 2012).

Nowaday, the sustainable development of the coffee industry faces severe challenges, particularly in the conservation of coffee germplasm resources and genetic diversity research (Van Der Vossen, 1985; Mekbib et al., 2022). As the foundation for breeding disease-resistant, stress-tolerant, and high-yield varieties, coffee germplasm resources are critical for addressing climate change, pest and disease threats, and diverse consumer demands (Mekbib et al., 2022). At present, there are many challenges in the conservation and utilization of coffee germplasm resources. (1) Habitat Loss: Over 30% reduction in wild coffee habitats globally, with climate change shrinking suitable cultivation areas by 1.2% annually (Moat et al., 2017; Bunn et al., 2015). Extreme weather events increasingly threaten coffee yield and quality (Bunn et al., 2015). (2) Biotic Stresses: Devastating outbreaks of coffee leaf rust (*Hemileia vastatrix*) and Coffee Berry Borer (*Hypothenemus hampei*) are exacerbated by declining genetic diversity, which weakens disease resistance (Avelino et al., 2015). (3) Genetic Erosion: Agricultural expansion and land-use changes have endangered wild coffee germplasm, pushing rare varieties toward extinction (Davis et al., 2021). These issues threaten both coffee industry sustainability and global supply chain stability (Pham et al., 2019). This rapid depletion of genetic options threatens to collapse the delicate balance between sustainable production and ecological preservation, potentially destabilizing a global supply chain that supports millions of livelihoods and satisfies evolving consumer demands for both quantity and quality (Moat et al., 2017). The stark reality is that without immediate, coordinated efforts to conserve and study remaining coffee genetic diversity, the industry risks being left defenseless against the combined onslaught of climate change, emerging pests and diseases, and shifting market requirements - challenges that diverse germplasm could help overcome if preserved and properly utilized (Bunn et al., 2015). This genetic diversity represents not just scientific interest but the very foundation upon which the coffee industry’s climate adaptation strategies, disease resistance breeding programs, and quality improvement initiatives must be built to ensure both economic sustainability and ecological balance for generations to come (Moat et al., 2017). Strengthening the conservation, research, and utilization of coffee germplasm is thus essential for ensuring long-term industry viability and ecological balance (Moat et al., 2017).

Ethiopia, recognized as the center of origin for *Coffea arabica*, preserves approximately 99% of its wild genetic diversity (Davis et al., 2021; Ceja-Navarro et al., 2015). In China, coffee cultivation dates back over a century. The country has established extensive germplasm collections and conducted preliminary evaluations of these resources. However, most studies to date have focused on phenotypic characterization, with limited exploration of genetic traits (Zhou et al., 2015). The Chinese Germplasm Repository of Coffee RuiLi City, Ministry of Agriculture and Rural Affairs, stands as the nation’s largest and most comprehensive coffee germplasm facility. It currently safeguards over 952 accessions, encompassing diverse cultivated varieties, wild relatives, and hybrid populations developed through both natural and artificial crosses. These materials provide a critical foundation for advancing genetic breeding research (Zhao et al., 2025). Core germplasm collections play a pivotal role in efficiently exploring and conserving genetic resources (Zhang et al., 2011). Such collections are carefully curated subsets of germplasm, designed to encapsulate maximum genetic diversity within a minimal sample size. This strategic approach serves as a cornerstone for effective germplasm management and utilization (Liu et al., 2020). Studies indicate that core collections typically represent 5% to 30% of total accessions, though this proportion varies across crops (Ndjiondjop et al., 2017). In coffee breeding, core collections offer a streamlined platform for identifying superior traits. For instance, breeders can rapidly screen for high yield, superior cup quality, disease resistance, or environmental stress tolerance. This efficiency significantly accelerates genetic improvement programs (Clifford and Willson, 1985; Vossen, 1985). However, existing coffee core collections—including those in Ethiopia and Brazil—face critical limitations. Many suffer from inadequate sampling (representing <15% of genetic diversity) or rely on low-resolution molecular markers (SSRs or 5K SNPs). These shortcomings hinder their ability to comprehensively capture genetic diversity (Gautam et al., 2004; Cunha Alves and Azevedo, 2018). To address these gaps, this study establishes a 185-accession Coffee Core Germplasm Collection (CCGC). The selection criteria prioritize geographic representation (Kenya, Polundi, Cote d ‘Ivoire, Colombia, Ethiopia, India, Portugal, etc), phenotypic diversity (disease resistance, productivity, flavor profiles), and historical contributions to breeding programs. The genomic representativeness of the CCGC is rigorously validated using Restriction-site Associated DNA sequencing (RAD-seq) technology.

Advances in biotechnology have established molecular techniques as the most reliable methods for germplasm characterization. RAD-seq offers a cost-effective genomic analysis approach. By utilizing restriction enzyme digestion or target-specific primers to enrich genomic regions of interest, this technique selectively sequences partial genomes, dramatically reducing both sequencing costs and data volume (Davey et al., 2011). RAD-seq efficiently generates single nucleotide polymorphisms (SNPs) and insertion-deletion (InDel) markers, making it ideal for large-scale genetic diversity studies (Peterson et al., 2012). Compared to whole-genome sequencing, RAD-seq maintains high resolution while significantly lowering costs and computational complexity, particularly advantageous for resource-limited species or projects (Elshire et al., 2011; Krishnan, 2022). In coffee germplasm research, RAD-seq enables rapid and economical identification of genetic variations, providing essential data for conservation and utilization (Krishnan, 2022). The evolution of next-generation sequencing technologies has made whole-genome sequencing more efficient and affordable than ever, unlocking opportunities to detect extensive DNA polymorphisms (Arai-Kichise et al., 2011). SNPs-the most prevalent genomic variations—refer to single-base substitutions (Bhattramakki et al., 2002). To date, SNP markers have become central to molecular assays due to their compatibility with high-throughput automated platforms (Jangra et al., 2021). Meanwhile, InDels (1–50 bp insertions or deletions) are gaining recognition for their growing importance in genetic variation (Salathia et al., 2007). Both markers are well-suited for genetic evaluation and selective breeding strategies using molecular genetics. Coffee genetic diversity studies face notable technical limitations. First, molecular marker applications remain oversimplified, with 82% of published studies relying solely on SSR markers (Yan et al., 2019). However, SSRs struggle to deliver precise analyses for large sample sets—a gap addressed by RAD-seq through its genome-wide high-throughput SNP coverage. For instance, the coffee single nucleotide polymorphism (SNP) chip developed by Cheng et al. contains only 5,000 loci, which is significantly lower than that of crops such as cocoa (30,000 SNPs) (Merot-L’anthoene et al., 2019; Guo et al., 2021). Secondly, the research on the core germplasm resources of coffee worldwide is still lagging behind. Currently, only Ethiopia and Brazil have established regional germplasm banks (Gautam et al., 2004; Cunha Alves and Azevedo, 2018). Yet these collections inadequately sample genetic diversity (<15% of total resources) and rely on outdated evaluation systems dominated by morphological markers (~30% of studies) and basic genetic parameters (e.g., Nei’s diversity index), lacking advanced genomic approaches like genome-wide association studies (GWAS) (Otyama et al., 2019). In China, Huang Lifang’s research on coffee diversity using RAPD technology is a pioneering work. This study not only analyzed the genetic relationships but also confirmed the applicability of this marker in variety identification (Huang et al., 2014, 2017, 2016). Parallel advances in other crops offer valuable insights: Li et al. integrated multiple SNP markers from whole-genome data of 117 rice accessions to construct a fingerprinting system that minimizes false positives/negatives and enables rapid identification (Li et al., 2020). Similarly, Peng et al. developed Indel markers for gene mapping, successfully characterizing rice male sterile lines (Liu et al., 2017). Despite the widespread adoption of molecular markers in germplasm evaluation, existing coffee core collections still lack genome-wide validation of their genetic representativeness. Based on the current technological development, reduced-representation sequencing has emerged as an ideal method for analyzing the genetic diversity of coffee due to its efficiency, economy and universality. This study addresses this critical knowledge gap by employing RAD-seq to systematically dissect genome-scale genetic diversity within coffee core germplasm resources.

This study aims to comprehensively analyze the genetic diversity of 185 coffee germplasm accessions using RAD-seq and validate the germplasm collection. For the first time, we systematically characterized the genome-wide genetic diversity of coffee core germplasm resources, providing a scientific foundation for coffee breeding and resource conservation. By applying RAD-seq technology, we identified 37,729 loci, achieving a marker density 7.5 times higher than that of existing coffee core collections (5K SNP array) and accomplishing the first genome-wide validation of genetic representativeness. The research not only fills the knowledge gap in genetic diversity studies of coffee germplasm resources in China but also offers scientific guidance for their classification, conservation, and innovative utilization. Furthermore, it provides an innovative solution for the global conservation and sustainable use of coffee genetic resources.

## 2 Materials and methods

### 2.1 Plant material

This study utilized 185 accessions of *Coffea arabica* L. germplasm maintained at the Chinese Germplasm Repository of Coffee RuiLi City, Ministry of Agriculture and Rural Affairs, representing three principal genetic groups (*Bourbon*/*Typica*, Ethiopian native, and Introgression group) collected from major coffee-producing countries including Kenya, Burundi, Côte d’Ivoire, Colombia, Ethiopia, India, and Portugal (**Supplementary Table S1**). The coffee samples were sourced from 7 major producing countries and exhibited significant geographical diversity. Stratified sampling by region was employed. The 952 samples were divided into 7 layers based on the country. The original number of samples in each layer was recorded, and the number of samples to be selected from each layer was calculated based on its proportion in the overall population. Then, simple random sampling was independently used to select the target number of samples from each layer. The total number of final samples in each layer was checked to be 185, ensuring no omissions or repetitions, and the geographical distribution was consistent with the overall population. The germplasm collection exhibits extensive geographic diversity, significant phenotypic variation, and high genomic representation, making it particularly suitable for investigating genetic diversity patterns, disease resistance traits, and quality improvement potential in *Arabica* coffee breeding programs.

### 2.2 DNA extraction and quality control

Genomic DNA was extracted using a modified CTAB method. Fresh leaf tissues were ground into powder using liquid nitrogen. CTAB extraction buffer (2% CTAB, 100 mM Tris-HCl, 20 mM EDTA, 1.4 M NaCl, 0.2% β-mercaptoethanol) was added, followed by incubation in a 65°C water bath for 1 hour. The mixture was extracted with chloroform-isoamyl alcohol (24:1) and centrifuged to collect the supernatant. An equal volume of isopropanol was added to precipitate DNA, which was then washed with 70% ethanol and dissolved in TE buffer.

The extracted DNA was quality-controlled through three approaches: integrity verification via agarose gel electrophoresis, purity assessment using Nanodrop (OD260/280 ratios between 1.8-2.2), and quantification using Qubit 3.0 fluorometer (concentration ≥50 ng/μL, total yield ≥2 μg). These quality parameters ensured the DNA met specifications for subsequent library construction.

### 2.3 Reduced-representation genome library construction and sequencing

The ddRAD-seq (Double Digest Restriction-site Associated DNA sequencing) protocol was performed following the standardized workflow by Peterson et al (Peterson et al., 2012).

#### 2.3.1 Genomic DNA digestion

High-frequency cutter MseI (5′-TTAA-3′) and low-frequency cutter SacI (5′-GAGCTC-3′) (New England Biolabs, Ipswich, MA, USA) were used for double-digestion of 200 ng high-quality genomic DNA (OD260/280 = 1.8-2.0) in 1× CutSmart Buffer with 0.5 U/μL enzyme concentration. Reactions were incubated at 37°C for 2 h to ensure complete digestion.

#### 2.3.2 Adapter ligation and purification

Digested DNA fragments were ligated to custom-designed double-stranded adapters (containing 8 bp sample-specific barcodes and Illumina P5/P7 sequencing adapters) using 1× T4 DNA Ligase Buffer (Thermo Fisher Scientific, Waltham, MA, USA), 0.5 μM adapters, and 400 U T4 DNA ligase (Thermo Fisher Scientific). Ligation proceeded at 16°C for 12 hr. Ligated products were purified using AMPure XP beads (Beckman Coulter, Brea, CA, USA) to remove unbound adapters and residual fragments.

#### 2.3.3 PCR amplification and size selection

Purified DNA was amplified for 18 cycles using KOD-Plus-Neo high-fidelity DNA polymerase (TOYOBO, Osaka, Japan) with a 65°C annealing temperature. Amplified products were separated on 1% low-melting-point agarose gels (Bio-Rad Certified Megabase Agarose in 1× TAE buffer), and fragments of 300–400 bp were excised and purified using the QIAquick Gel Extraction Kit (QIAGEN, Hilden, Germany).

#### 2.3.4 Library quality control

Purified libraries were quantified using a Qubit 3.0 Fluorometer (Thermo Fisher Scientific; ≥2 nM). Insert size distribution (300–400 bp, CV <5%) was validated on an Agilent 2100 Bioanalyzer (Agilent Technologies, Santa Clara, CA, USA). Effective library concentration (≥2 nM) was determined via KAPA Library Quantification Kit (Roche, Basel, Switzerland) on an ANALYTIKJENA qTOWER real-time PCR system (Jena, Germany).

#### 2.3.5 High-throughput sequencing

Qualified libraries were pooled at equimolar ratios and sequenced on an Illumina HiSeq X Ten platform (Illumina, San Diego, CA, USA) with 150 bp paired-end (PE150) reads. Each sample generated an average of 4.7 Gb raw data (Q30 ≥94.25%). Raw sequencing data in FASTQ format retained sample-specific barcodes for downstream demultiplexing.

### 2.4 Bioinformatics analysis

#### 2.4.1 Data quality control and filtering

The raw sequencing data were processed using fastp (version 0.23.0) for quality control with the following parameter settings: adapter sequences were automatically detected and trimmed using the default adapter library; low-quality bases were filtered using a sliding window approach with a window size of 4 bp, trimming regions where the average Phred quality score fell below 15; and only reads ≥50 bp in length were retained. The resulting high-quality clean reads were used as the basis for subsequent analysis.

#### 2.4.2 Sequence alignment

Quality-controlled reads were aligned to the coffee reference genome (ET-39, NCBI GenBank: GCA_036785885.1) using BWA-MEM (version 0.7.15) with default parameters (Salojärvi et al., 2024). The alignment results were output as SAM files, which were subsequently converted to BAM format, sorted, and indexed using samtools (version 1.3.1) to facilitate variant calling.

#### 2.4.3 Variant detection

Genetic variant identification was conducted following the Genome Analysis Toolkit (GATK; version 3.7) workflow. The analysis pipeline comprised three sequential steps: (1) individual variant calling was performed using the HaplotypeCaller algorithm in GVCF mode, (2) joint genotyping across all samples was executed with the GenotypeGVCFs module, and (3) stringent quality filtering was applied. For SNP variants, we implemented the following filtering criteria: QD < 2.0, FS > 60.0, MQ < 40.0, SOR > 3.0, MQRankSum < -12.5, or ReadPosRankSum < -8.0. Indel variants were filtered using: QD < 2.0, FS > 200.0, ReadPosRankSum < -20.0, or SOR > 10.0. Additional population-level quality thresholds included: minimum read depth (DP) ≥ 3, genotype missing rate ≤ 50%, minor allele frequency (MAF) ≥ 5%, and maximum sample heterozygosity ≤ 60%. The final high-confidence variant set was output in VCF format for subsequent population genetic analyses.

#### 2.4.4 Variant annotation

Detected variants were functionally annotated to characterize: SNP types (transitions/transversions), InDel length distribution, Genomic density and distribution patterns. Annotation results were saved in ANN format, providing comprehensive variant information for downstream population genetic analyses.

#### 2.4.5 Population genetic analyses

Multiple approaches were employed to validate the global coffee genetic diversity coverage of the germplasm collection and assess its genetic structure rationality.

#### 2.4.6 Population structure analysis

Genetic structure was inferred using STRUCTURE (version 2.3.4) with K-values ranging from 2 to 10. Ten independent runs were performed for each K using the default parameters: a burn-in period of 10,000 iterations followed by 100,000 Markov chain Monte Carlo (MCMC) replications under the admixture model. The optimal K-value was determined using the ΔK method, and output files were processed to visualize subpopulation clustering patterns.

#### 2.4.7 Principal component analysis

Genetic differentiation among populations was assessed using PLINK (version 1.9). A covariance matrix derived from genotype data was subjected to eigenvalue decomposition, and principal components were computed. The PCA results were stored in EIGENSTRAT format, enabling multidimensional scaling visualization of genetic relationships between populations.

#### 2.4.8 Phylogenetic tree construction

The phylogenetic analysis was performed using the neighbor-joining (NJ) method in MEGA11 with the following parameters: (1) genetic distances were calculated using the Kimura 2-parameter (K2P) substitution model, which was selected based on the lowest Bayesian Information Criterion (BIC) score in MEGA’s model test module; (2) rate variation among sites was modeled using a gamma distribution (shape parameter = 1.0) with invariant sites; and (3) nodal support was assessed with 1,000 bootstrap replicates. The NJ tree construction was conducted with pairwise deletion of gaps/missing data. The final tree topology, including branch lengths and bootstrap values, was exported in Newick format for evolutionary relationship analysis among coffee populations.

#### 2.4.9 Genetic diversity and differentiation analysis

Genetic diversity parameters—including observed heterozygosity (Ho), expected heterozygosity (He) and nucleotide diversity (π) were computed using Arlequin (version 3.5). Analysis outputs provided quantitative metrics for assessing population-level genetic variation and divergence across subgroups.

### 2.5 Statistical analysis

All analytical results were statistically processed and visualized using R (version 4.2.1) and Python (version 3.9) scripts, encompassing data quality control, variant distribution profiling, and population structure characterization. Visualization workflows were implemented via R packages including ggplot2, pheatmap, and circlize, generating publication-ready figures in PDF and PNG formats. Code repositories and parameter settings were archived to ensure analytical accuracy and reproducibility of findings.

## 3 Results

### 3.1 RAD-seq data quality control and filtering

The distribution characteristics of base composition (A, T, C, G, N) in RAD-seq data serve as crucial indicators for assessing sequencing data quality. During library preparation and sequencing processes, factors such as PCR amplification bias may lead to A/T and G/C separation phenomena, potentially compromising data accuracy and reliability. According to sequencing principles and the principle of complementary base pairing, GC content and AT content should remain relatively stable across each sequencing cycle under ideal conditions, maintaining a consistent horizontal distribution trend throughout the sequencing process. Notably, the proportion of N bases (representing unidentifiable base types) serves as a key reference metric for evaluating sequencing quality.

The sequencing base composition distribution results for this project are shown in **Supplementary Figure S1**. Due to the connection with primer adapters at sequencing initiation sites, A, C, G, and T contents exhibited initial fluctuations at starting positions. However, these base compositions gradually stabilized as sequencing progressed. Particularly noteworthy is that the proportion of unknown bases (N) remained consistently low throughout the process. This observation indicates minimal systemic AT bias during sequencing and reflects that both library construction quality and sequencing performance met optimal standards, satisfying the requirements for subsequent bioinformatics analyses.

Statistical analysis of clean data from sequencing 185 coffee germplasm resources (**Supplementary Table S2**). A total of 216,178,439,423 raw base pairs were obtained. Each sample yielded an average of 4,700,228 clean paired-end (PE) reads. The average sequencing output was 4.7 Gb raw data (Q30 ≥ 94.25%). The mean Q20 value (base recognition accuracy ≥ 99%) was 97.89%, and the mean Q30 value (base recognition accuracy ≥ 99.9%) was 94.25%, with an average GC content of 42.16%. These results demonstrate high base-calling efficiency, excellent sequencing quality, and realistic GC distribution. The data quality meets the requirements for ddRAD-seq analysis and is suitable for downstream bioinformatics analyses.

### 3.2 Data comparison rate and coverage statistics

To further investigate the genomic characteristics of the 185 coffee germplasms, sequencing data were aligned to the coffee reference genome (ET-39 v2.4) using BWA software. The alignment rate reflects the similarity between the sample genome and the reference genome. If the reference genome is appropriately selected and no contamination occurred during experiments, the alignment rate of paired-end (PE) reads should exceed 70%. In this study, 88.77% of sequencing data from all samples aligned to the reference genome on average (**Supplementary Table S3**), indicating normal library construction and absence of contamination.

Genome coverage refers to the percentage of the reference genome that the read sequences cover, reflecting the completeness of variant detection. Coverage depth refers to the average number of reads covering each base, which affects the accuracy of variant detection. After mapping the read sequences to the reference genome, analysis was conducted on these two key indicators. The results showed that for 185 coffee germplasm, the average genome coverage was 3.05% and the average coverage depth was 17.66× (**Supplementary Figure S2**; **Supplementary Table S4**).

### 3.3 Variation type detection and distribution

**Figure 1:**
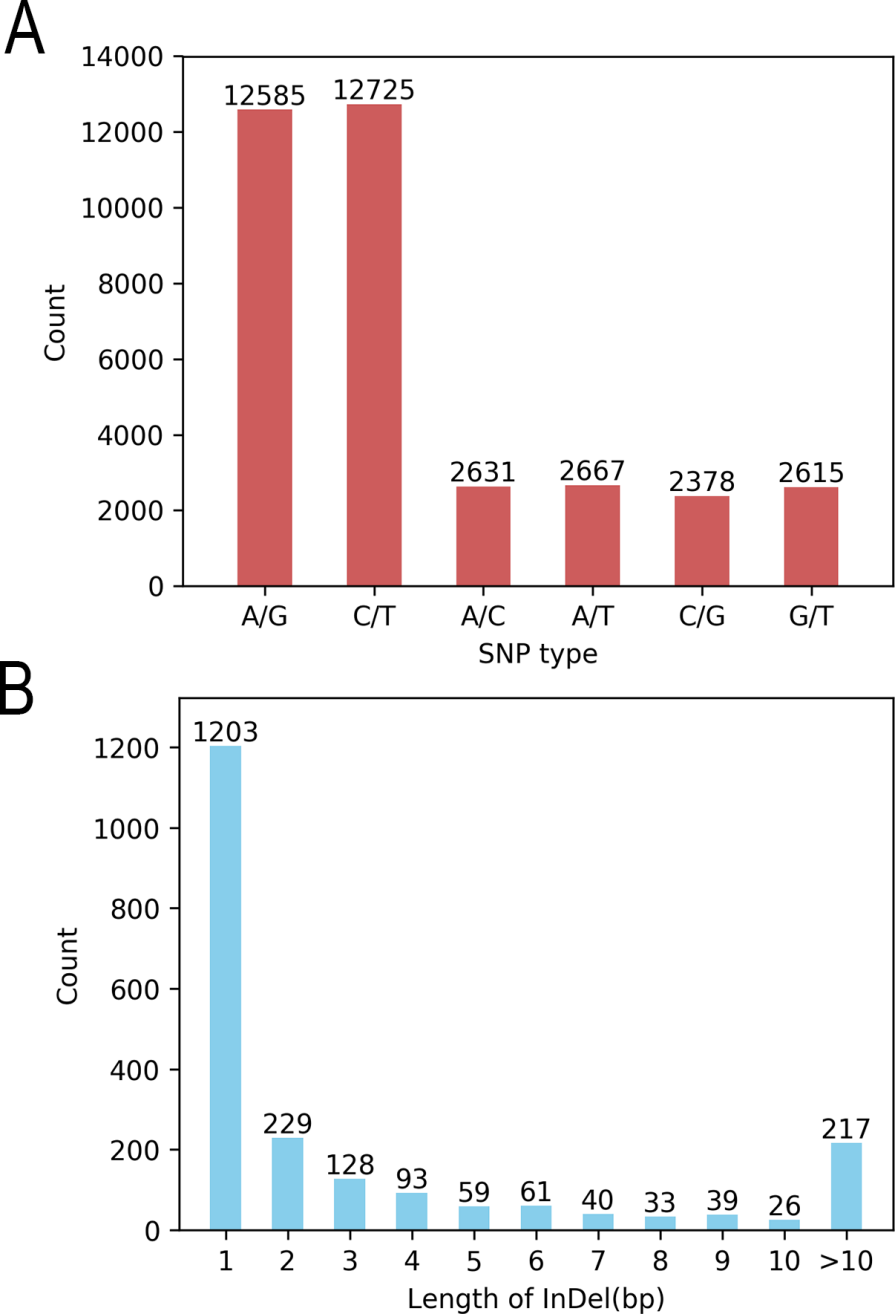
Statistics of variation types. **(A)** SNP conversion/transposition types and **(B)** InDel length distribution.

SNP detection was performed on the 185 coffee germplasms using GATK software, yielding 37,729 variant sites, including 35,601 SNPs and 2,128 InDels (**Table 1**, **Figure 1**). Among the SNPs, 25,310 were transitions (A/G and C/T), and 10,291 were transversions (A/C, A/T, C/G, and G/T), with a transition-to-transversion ratio (Ts/Tv) of 2.46. The distribution of SNP types and length statistics of InDels are shown in the **Figure 1**. Significant differences were observed in the number and density of variants across chromosomes, correlating with chromosome length, gene distribution, and functional regions.

**Table 1:** Types and density of variation.

Chr	Length	No. SNPs	SNP density	No. InDels	InDel density
CA1	59,034,463	4,705	79.70	269	4.56
CA2	57,716,770	734	12.72	64	1.11
CA3	76,906,396	1,708	22.21	111	1.44
CA4	77,301,657	1,045	13.52	73	0.94
CA5	44,587,295	2,058	46.16	153	3.43
CA6	49,228,674	738	14.99	32	0.65
CA7	52,323,663	4,063	77.65	226	4.32
CA8	48,754,064	392	8.04	17	0.35
CA9	49,172,495	1,577	32.07	101	2.05
CA10	55,030,746	543	9.87	28	0.51
CA11	63,716,479	3,389	53.19	168	2.64
CA12	63,214,365	679	10.74	51	0.81
CA13	40,515,400	3,157	77.92	215	5.31
CA14	50,646,195	465	9.18	39	0.77
CA15	46,623,164	1,495	32.07	109	2.34
CA16	53,737,150	874	16.26	41	0.76
CA17	44,254,144	3,175	71.74	163	3.68
CA18	41,449,412	595	14.35	38	0.92
CA19	54,177,763	753	13.90	46	0.85
CA20	48,872,521	365	7.47	22	0.45
CA21	45,802,622	2,497	54.52	130	2.84
CA22	62,661,540	594	9.48	32	0.51
Whole	1,185,726,978	35,601	30.02	2,128	1.79

Chr, Chromosome number; Length, chromosome length; No. SNPs, indicates the number of SNPS; SNP density, SNP density (number of SNPS per Mb); No. InDels, The number of InDel; InDel density, Density of InDel (number of InDel per Mb).

This study analyzed the genomic distribution of variants, revealing significant heterogeneity in variant counts and densities across chromosomes. For SNPs, CA1 exhibited the highest count (4,705), while CA8 had the lowest (392), with a genome-wide total of 35,601 SNPs (CA refers to chromosome number). The SNP density ranged from 79.70 SNPs per Mb on CA1 to 7.47 SNPs per Mb on CA20, averaging 30.02 SNPs per Mb genome-wide. For InDels, CA1 showed the highest number (269), whereas CA2 had fewer (64), with a total of 2,128 InDels genome-wide. InDel density varied from 4.56 InDels per Mb on CA1 to 0.45 InDels per Mb on CA20, with an average genome-wide density of 1.79 InDels per Mb. These findings confirm the non-uniform distribution of variants, with striking differences in SNP/InDel abundance and density among chromosomes. This variability provides critical insights for investigating genome structure, functional regions, and the biological implications of genetic variation.

### 3.4 Population structure analysis

**Figure 2:**
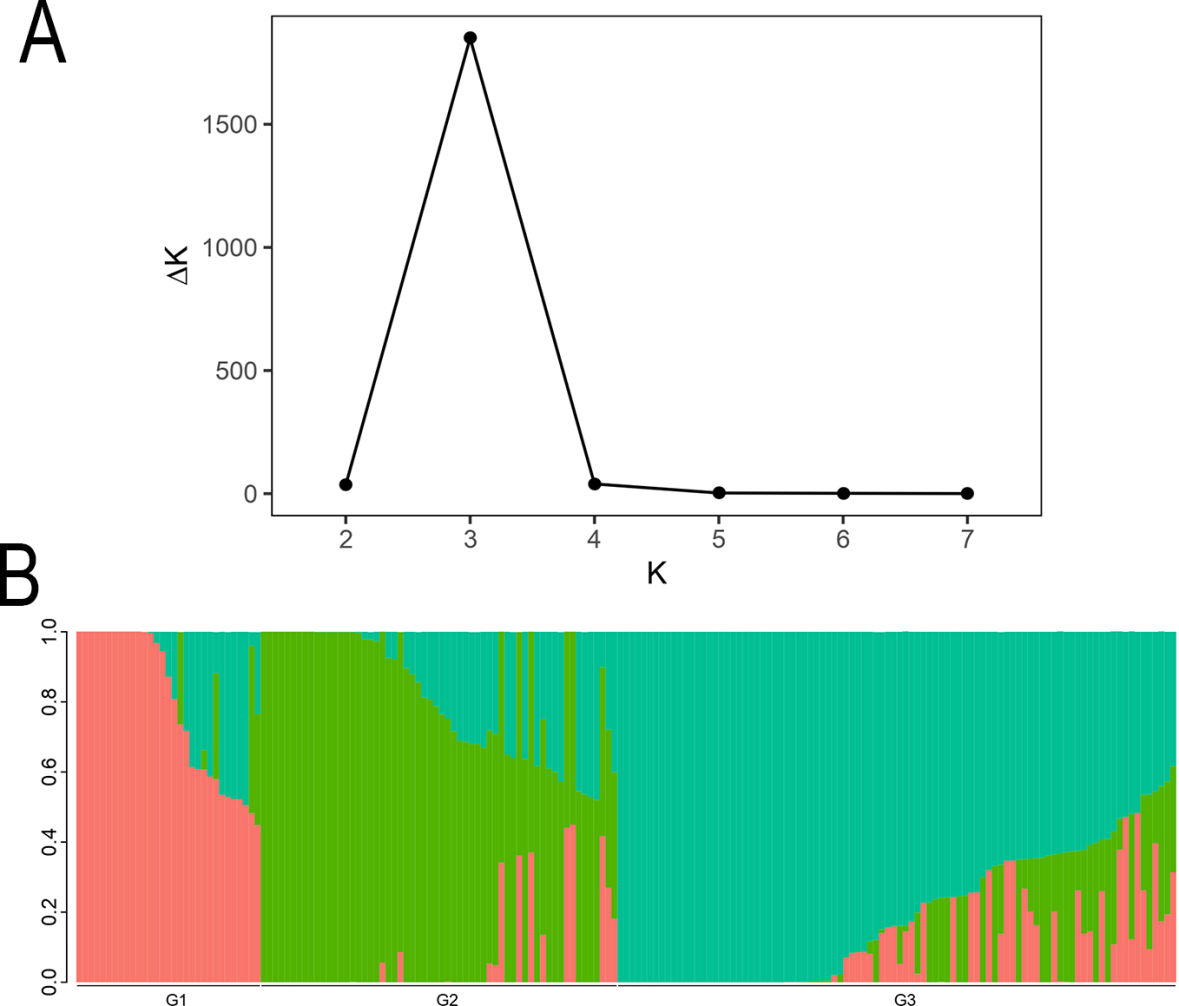
The Structure analysis of 185 coffee germplasm resources. **(A)** The change trend chart of ΔK. **(B)** Histogram of Q value of each sample when K=3. In the Structure diagram, each color represents a subgroup. A stacked column chart in the diagram represents an individual’s “ancestry”, and an individual with only one color indicates a relatively pure lineage, while those with multiple colors indicate a mixed lineage. Through the colors, we can divide the individuals in the population into different subgroups.

Based on the STRUCTURE analysis of 185 coffee germplasm resources, the optimal number of subgroups was determined to be K=3 (corresponding to the highest ΔK value), indicating that the genetic structure of the tested coffee population is most distinct when divided into three groups (**Figure 2**). The ΔK trend and the distribution of individuals within subgroups are illustrated in the **Figure 2**. The population genetic analysis revealed that the 185 coffee accessions are best classified into three groups: Group 1 contains 31 coffee varieties, Group 2 includes 60 coffee varieties, and Group 3 comprises 94 coffee varieties.

**Figure 3:**
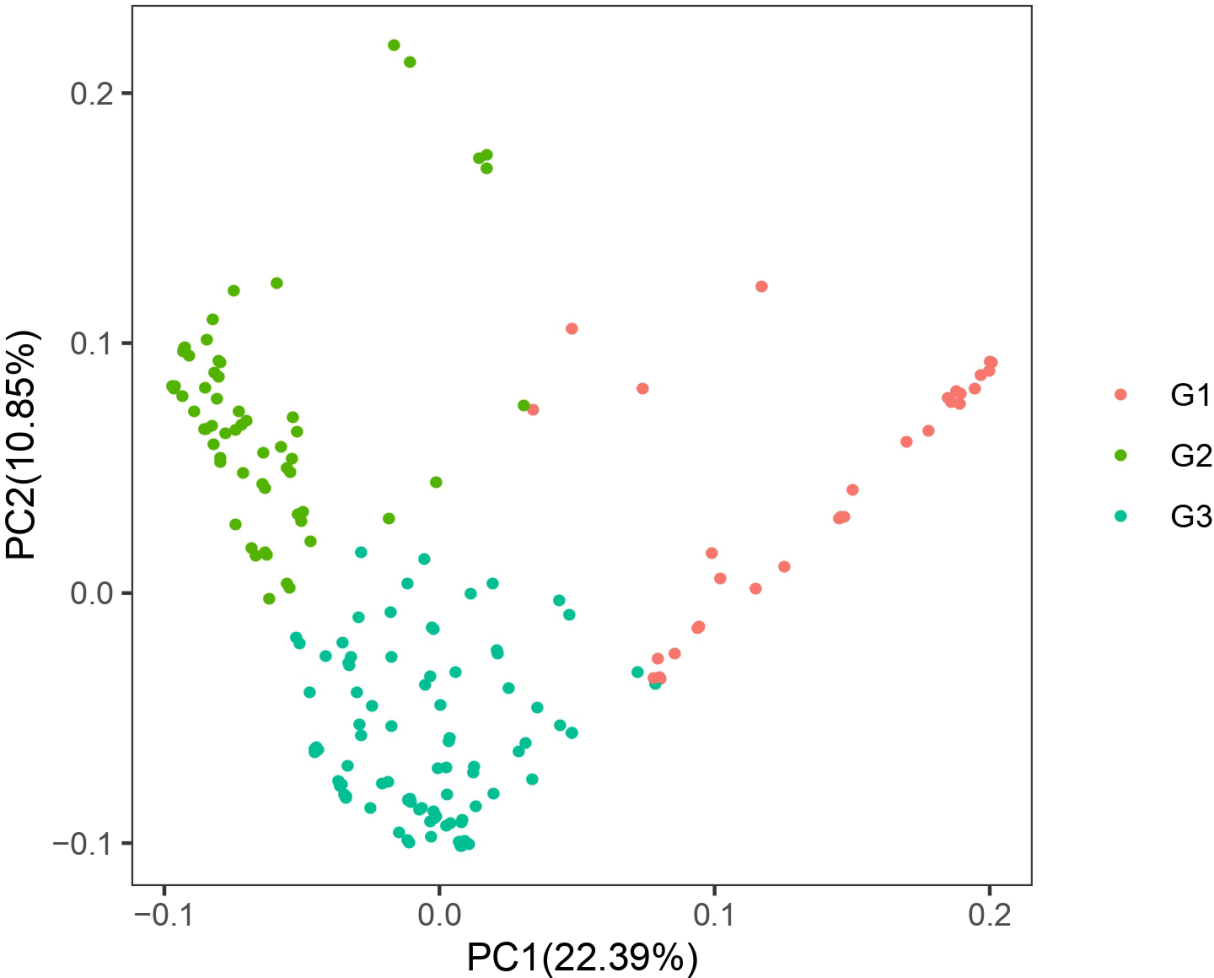
Scatter plot of PCA of 185 coffee germplasm resources.

PCA (**Figure 3**) of the 185 coffee germplasm resources was performed using PLINK based on SNP differences among individual genomes. The results, categorized into three genetic groups, aligned with the earlier population structure analysis, further supporting the hypothesis of substantial genetic diversity among the 185 coffee accessions.

**Figure 4:**
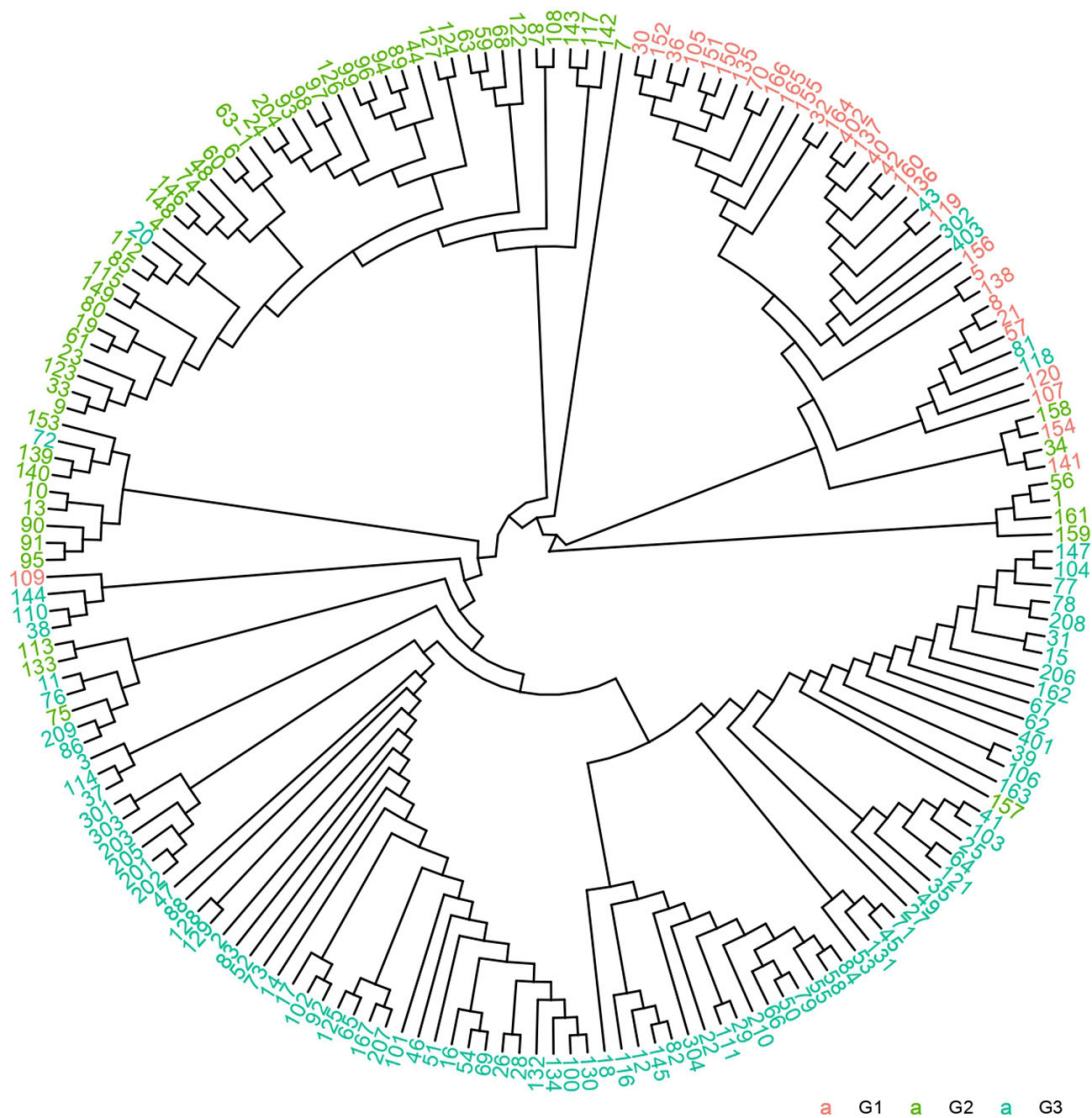
Phylogenetic tree of 185 coffee germplasm resources.

A phylogenetic tree was constructed using the NJ method based on genetic distances derived from SNP markers (**Figure 4**). Samples were labeled according to their prior group classifications. Intriguingly, coffee varieties from different groups were observed within the same clades. To validate these findings, the phylogenetic tree was integrated with population structure analyses, incorporating scenarios for K=2, 3, 4, and 5. The combined results demonstrated that K=3 remained the optimal grouping despite minor overlaps of certain varieties in shared clades, consistent with the population structure conclusions. This further reinforces the hypothesis of pronounced genetic divergence among the 185 accessions. The phylogenetic tree revealed that the 185 germplasm samples spanned all 11 major evolutionary clades of the Coffea genus. The genetic diversity parameter π = 0.1456 confirms the representativeness of the 185 coffee variety samples.

### 3.5 Genetic diversity analysis and genetic differentiation analysis

Genetic diversity, defined as the genetic variation among different populations or individuals within a species, serves as the foundation for survival, adaptation, and evolution. In this study, expected heterozygosity (He), polymorphism information content (PIC), and nucleotide diversity (π) were used to assess the genetic diversity of each subgroup. Genetic diversity metrics were calculated using VCFtools with a window size of 100 kb and a step size of 20 kb.

The results show G2 exhibited the highest values across all three metrics, indicating relatively greater genetic diversity compared to G1 and G3 (**Table 2**). In contrast, G3 showed the lowest nucleotide diversity (π). We hypothesize that the elevated diversity in G2 may reflect its inclusion of more wild genetic resources or divergent selection pressures compared to the other groups.

**Table 2:** Statistical table of genetic diversity indicators.

Group	He	PIC	π
G1	0.1432	0.1205	0.1326
G2	0.3014	0.2428	0.1456
G3	0.1633	0.1425	0.1031

He, expected heterozygosity; PIC, polymorphism information content; π, nucleotide diversity.

**Figure 5:**
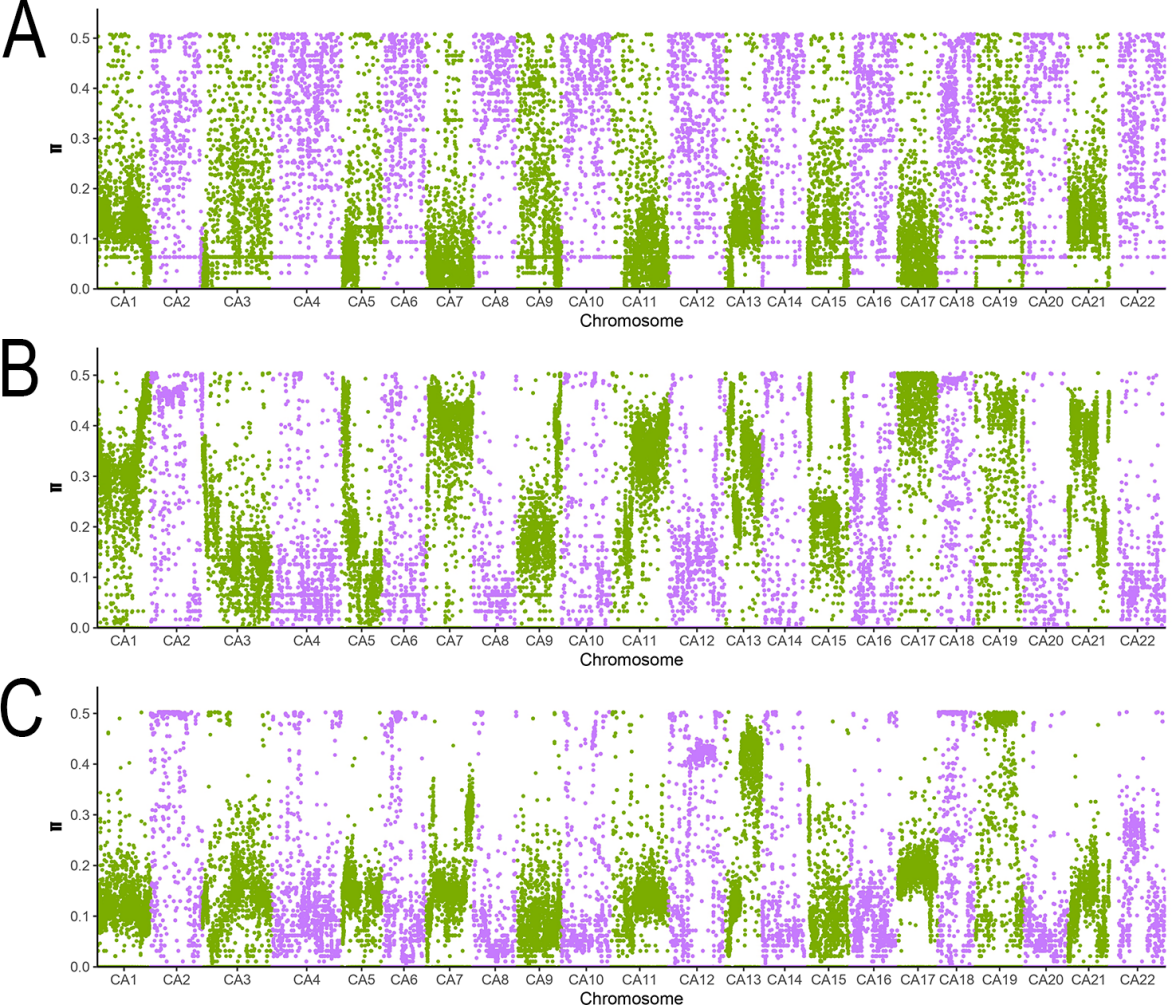
Distribution of whole-genome π values of the three subpopulations. This figure presents the distribution of nucleotide diversity (π value) across 22 chromosomes (CA1-CA22) in the three groups of samples through STRUCTURE analysis. ABC represent Group 1, Group 2, and Group 3 respectively. The purple and green data points are only used to distinguish adjacent chromosomes (to avoid visual confusion) and have no biological significance. CA1-CA22 represent chromosome numbers, and the vertical coordinate π value range (0.0 - 0.5) reflects the level of genetic diversity.

**Figure 5** illustrates the genome-wide distribution of π values across the three groups, providing a visual overview of their genetic diversity patterns. These findings offer robust data-driven insights into the genetic diversity of the germplasm resources, supporting their conservation and utilization.

## 4 Discussion

### 4.1 Analysis based on RAD-Seq confirmed the genetic diversity of Chinese coffee germplasm resources

Molecular markers (such as RFLP, SSR, SNP, etc.) are widely used in population genetics, and the advent of next-generation sequencing technology has significantly enhanced their development and application efficiency (Grover and Sharma, 2016; Huang et al., 2009). The application of molecular markers in coffee aligns with this trend and effectively reveals the genetic differentiation within coffee. Anthony et al. revealed the genetic diversity of Arabica coffee through AFLP and SSR markers, further supporting the importance of wild germplasm resources in coffee breeding (Anthony et al., 2002). Yunita et al. conducted a genetic diversity analysis of Arabica coffee in the Solok Regency region using the SRAP molecular marker technique (Yunita et al., 2020). The results showed that the He among the samples ranged from 0.2812 to 0.3638, indicating that the SRAP markers could effectively reveal the genetic variation characteristics of Arabica coffee in this region. Huang Lifang and others conducted a genetic diversity analysis on 87 coffee germplasm resources using RAPD primers (Huang et al., 2017). This provided important molecular basis for the evaluation and breeding of coffee germplasm resources. However, it should be noted that this study had a limitation of a small sample size, which might have affected the representativeness and statistical power of the research results to some extent. Based on the above research background, this study employed high-density SNP marker technology to conduct a systematic genetic diversity analysis on 185 coffee germplasm resources from China. The research results showed that the genetic diversity index (He) of Chinese coffee germplasm resources was 0.3014, which was similar to the results (H = 0.2812 - 0.3638) of Yunita et al.’s study on Arabica coffee in the Solok Regency area (Yunita et al., 2020). It is worth noting that both studies classified the test materials into three genetic groups. This finding further verified that Chinese coffee germplasm resources have moderate levels of genetic diversity. Moreover, the results of this study also confirmed that RAD-Seq technology, as an efficient SNP genotyping method for the entire genome, has comparable application value in crop genetic diversity analysis to SRAP markers.

### 4.2 Classification of genetic populations of 185 coffee varieties

The population structure of small-grain coffee (*Coffea arabica*) can be classified into three main groups based on its botanical characteristics and genetic evolution relationships: the Bourbon/Typica cultivar group, the Ethiopian Native original species group, and the Introgression Group hybrid population (Salojärvi et al., 2024). This classification system not only reflects the evolutionary history of coffee populations, but also demonstrates the significant differences in agronomic traits and flavor characteristics among different groups. In this study, the results of molecular marker analysis indicated that 185 coffee germplasm resources could be clearly classified into three genetic groups (**Figures 2**–**4**). The results of population structure analysis, principal component analysis, and phylogenetic tree construction were all consistent in supporting this genetic grouping pattern, thereby verifying the classification hypotheses made in the previous stage of the research. These findings align with previous research, underscoring the rich genetic variation preserved in coffee germplasm resources worldwide (Aerts et al., 2013). This diversity provides a critical genetic foundation for coffee breeding programs and germplasm conservation efforts, enabling the identification of valuable traits for crop improvement and resilience against environmental challenges (Yan et al., 2019; Aerts et al., 2013). Genetic population analysis indicates that the G1 population may mainly consist of cultivated varieties from specific geographical regions, while the G2 population is significantly enriched with wild germplasm resources. However, there is a clear phenomenon of gene exchange between the populations, manifested by the migration of some germplasms between the G1 and G2 populations. This phenomenon of genetic component mixture may result from the following factors: (1) gene infiltration caused by artificial hybridization breeding (Ogutu, 2019). (2) adaptive evolution under natural selection pressure (Wan and Wootton, 2000). (3) selective elimination effects in specific genomic regions (Huang et al., 2017). In particular, individuals at the population boundary may carry special recombinant haplotypes, and these variations may be the key factors contributing to gene flow between the populations. For example, Sample number 154. This material was formed through the hybridization of CATURI and HDT, and it belongs to the gene infiltration group. However, since its parent strain HDT belongs to the native species of Ethiopia, it was classified into Group 1. For instance, the population differentiation phenomenon discovered by Iqbal et al., as well as the geographical isolation effect revealed by Pagani et al., all corroborate the conclusions of this study (Iqbal et al., 2022; Pagani et al., 2012).

### 4.3 Significance and optimization of core seed bank

A core germplasm collection is a critical strategy for efficient management and utilization of genetic resources, aiming to condense large-scale germplasm into a representative, genetically diverse subset through scientific screening and optimization (Yang et al., 2022). This collection serves as a high-efficiency platform for coffee breeding, enabling rapid identification of germplasm with desirable traits and shortening breeding cycles. Through the establishment of a core germplasm resource library for rice, they significantly improved the breeding efficiency, providing an important model for the field of crop genetic improvement (Zhang et al., 2011). Additionally, Liu et al. demonstrated the utility of core collections in resource management by integrating genetic and metabolic data to build a medicinal plant core collection (Liu et al., 2020). The marker density of this study (37,729 SNPs) is significantly higher than that of the reported rice core germplasm resources (12,000 SNPs), the existing coffee SNP chip technology (8,500 SNPs), and the cocoa core germplasm resources (30,000 SNPs) (Zhang et al., 2011; Merot-L’anthoene et al., 2019; Motamayor et al., 2013).

While the Coffee Core Germplasm Collection (CCGC) already encompasses global genetic lineages and functional genes, future efforts could enhance its utility by integrating phenotypic data (e.g., disease resistance, flavor metabolites) to improve genetic diversity and representativeness. Second, combining phenotypic and genomic data would enable more precise screening to ensure inclusion of germplasm with key agronomic traits. Furthermore, the digital management of germplasm resources (such as establishing a coffee germplasm resource database) will significantly enhance the efficiency of researchers and breeders in accessing and utilizing relevant resources. For instance, Ndjiondjop et al. effectively optimized the utilization strategy of germplasm resources by establishing a small core germplasm bank for rice. Their method can provide important references for the screening and integration of coffee core germplasm (Ndjiondjop et al., 2017).

### 4.4 Limitations and prospects of the study

Although this study has made significant progress, it still has certain limitations. In terms of sample selection, although the sample size is large, it mainly focuses on the two main cultivated varieties of coffee, and the coverage of wild varieties is insufficient. Future research needs to include more wild resources to comprehensively analyze the genetic diversity of coffee. In terms of research methods, although RAD-seq has cost-effectiveness advantages, its detection range is limited compared to whole-genome sequencing (WGS), and it may miss important genetic variations. It is recommended that subsequent research combine WGS technology, such as the whole-genome resequencing strategy adopted by Mekbib et al., to construct a more complete genetic map. This study provides an important reference framework for the analysis of coffee genetic diversity (Mekbib et al., 2022).

Future research directions may include: Functional exploration of disease/resistance genes and identification of genetic markers linked to key agronomic traits to expand candidate gene pools for molecular breeding. GWAS integrating phenotypic and genomic data to uncover loci associated with yield, quality, and disease resistance. Expanding the core germplasm collection by adding representative accessions to enhance its genetic diversity and utility.

## 5 Conclusions

This study systematically analyzed and validated the genetic diversity of 185 coffee germplasm resources using RAD-seq technology. The research results show that this sample set comprehensively covers all 11 major evolutionary lineages of the Coffee genus. Its genetic diversity parameters are π = 0.1456 and He = 0.3014. Population structure analysis (K=3) further confirmed its genetic representativeness. The marker density (37,729 SNPs) represents a 7.5-fold improvement over existing coffee core collections (5K SNP array) and surpasses comparable studies on crops such as rice (12K SNPs) and cacao (30K SNPs).

As the first genome-wide validation of genetic diversity in a coffee core germplasm collection, this research addresses a critical knowledge gap and provides a scientific foundation for precise classification, efficient conservation, and molecular breeding of coffee genetic resources. The establishment of this core collection not only creates a standardized platform for rapid screening of disease-resistant, stress-tolerant, and high-quality germplasm, but also serves as a crucial genetic reservoir for addressing climate change and pest/disease challenges.

## Data Availability

The original contributions presented in the study are publicly available. This data can be found here: https://www.ncbi.nlm.nih.gov/bioproject/PRJNA1309331/.
