# Supplementary material for: Population structure and genetic diversity of a coffee germplasm collection in China revealed by RAD-seq
Source: Front Plant Sci. 2025 Sep 4;16:1629553. doi: 10.3389/fpls.2025.1629553 (PMC12443757; doi:10.3389/fpls.2025.1629553)
Supplement: Supplementary file 1 [file DataSheet1.zip › Supplementary Materials/Figure S1. Base composition distribution of RAD-seq sequencing data from 185 coffee germplasms..docx]

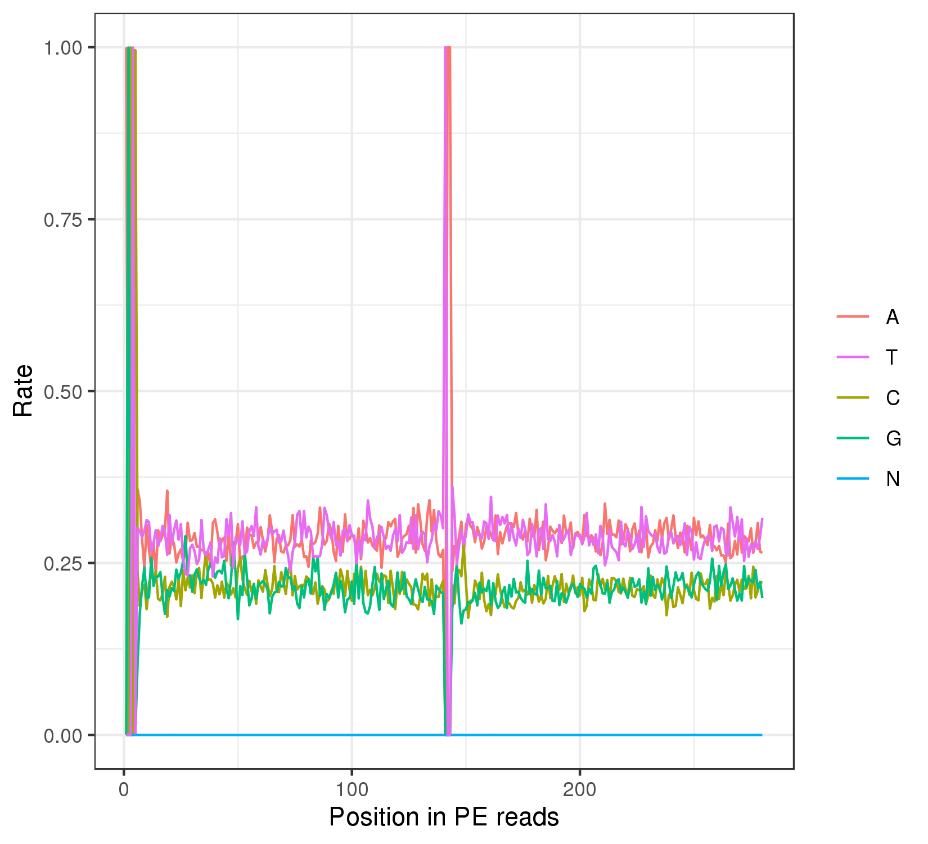


Figure S1. Base composition distribution of RAD-seq sequencing data from 185 coffee germplasms.

The x-axis represents the base position along reads, indicating the sequential arrangement of bases from the 5' to 3' end of the reads. The y-axis represents the percentage of each base (A, C, G, T, N) at each sequencing position across all reads, where N denotes undetermined base types. Different bases are represented by distinct colors.
