# Supplementary material for: Population structure and genetic diversity of a coffee germplasm collection in China revealed by RAD-seq
Source: Front Plant Sci. 2025 Sep 4;16:1629553. doi: 10.3389/fpls.2025.1629553 (PMC12443757; doi:10.3389/fpls.2025.1629553)
Supplement: Supplementary file 1 [file DataSheet1.zip › Supplementary Materials/Figure S2. Sequencing coverage and coverage depth distribution map of sequencing data on 22 chromosomes..docx]

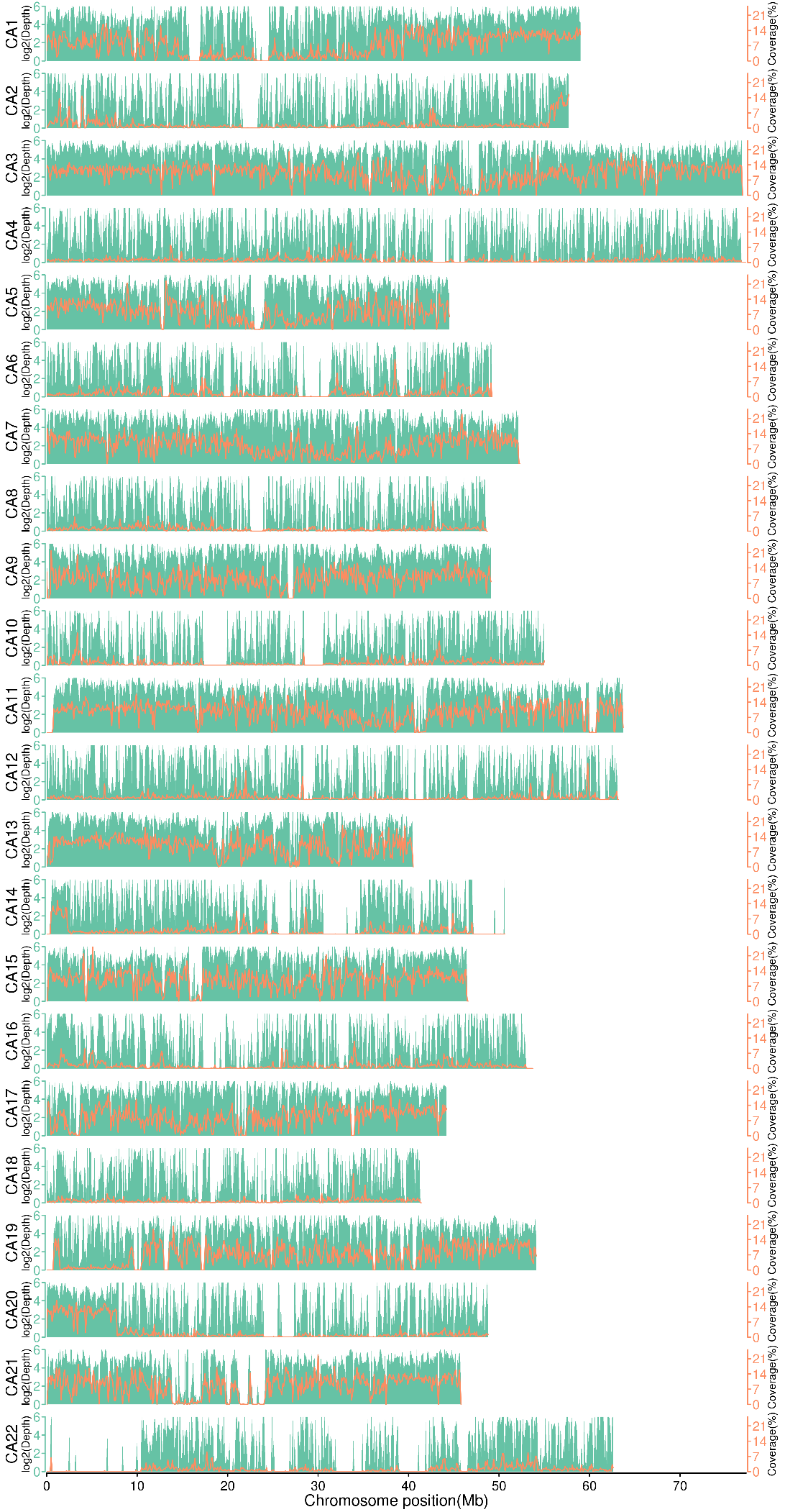


**Figure S2. Sequencing coverage and coverage depth distribution map of sequencing data on 22 chromosomes**. The green peak shows the sequencing depth. The yellow line shows sequencing coverage.
