# Supplementary material for: Population structure and genetic diversity of a coffee germplasm collection in China revealed by RAD-seq
Source: Front Plant Sci. 2025 Sep 4;16:1629553. doi: 10.3389/fpls.2025.1629553 (PMC12443757; doi:10.3389/fpls.2025.1629553)
Supplement: Supplementary file 1 [file DataSheet1.zip › Supplementary Materials/Table S1. The names and species of 185 coffee germplasm resources..docx]

**Table S1. The names and species of 185 coffee germplasm resources.** Group is the result of PCA analysis in Result 3.4.

| **Sample number** | **Variety name** | **Variety type** | **Group** |
| --- | --- | --- | --- |
| 1 | DTARI18 | Gene infiltration group | G2 |
| 2 | DTARI19 | Bourbon/Typica group | G3 |
| 3 | DTARI23-4 | Bourbon/Typica group | G3 |
| 4 | DTARI38 | Gene infiltration group | G2 |
| 5 | DTARI40 | Ethiopian native group | G1 |
| 6 | DTARI47 | Gene infiltration group | G2 |
| 7 | DTARI51 | Gene infiltration group | G2 |
| 8 | DTARI105 | Bourbon/Typica group | G1 |
| 9 | DTARI296 | Gene infiltration group | G2 |
| 10 | DTARI29 | Gene infiltration group | G2 |
| 11 | DTARI132 | Gene infiltration group | G3 |
| 12 | DTARI152 | Gene infiltration group | G3 |
| 13 | DTARI153 | Gene infiltration group | G2 |
| 14 | DTARI155 | Gene infiltration group | G3 |
| 15 | DTARI205 | Bourbon/Typica group | G3 |
| 16 | DTARI301A | Bourbon/Typica group | G3 |
| 17 | DTARI316 | Bourbon/Typica group | G3 |
| 18 | DTARI333 | Bourbon/Typica group | G3 |
| 19 | DTARI48 | Gene infiltration group | G2 |
| 20 | DTARI361 | Gene infiltration group | G3 |
| 21 | DTARI369 | Ethiopian native group | G1 |
| 22 | DTARINB1 | Gene infiltration group | G3 |
| 23 | DTARI389 | Gene infiltration group | G2 |
| 24 | DTARI398 | Gene infiltration group | G2 |
| 25 | DTARI400 | Gene infiltration group | G3 |
| 26 | DTARI404 | Bourbon/Typica group | G3 |
| 27 | DTARI408 | Unknown | G3 |
| 28 | DTARI409 | Bourbon/Typica group | G3 |
| 29 | DTARI424 | Gene infiltration group | G3 |
| 30 | DTARI476 | Ethiopian native group | G1 |
| 31 | DTARI518 | Ethiopian native group | G3 |
| 32 | DTARI537 | Ethiopian native group | G1 |
| 33 | DTARI538 | Gene infiltration group | G2 |
| 34 | DTARI585 | Gene infiltration group | G2 |
| 35 | DTARI411 | Bourbon/Typica group | G3 |
| 36 | DTARI413 | Ethiopian native group | G1 |
| 37 | DTARI414 | Bourbon/Typica group | G3 |
| 38 | DTARI420 | Gene infiltration group | G3 |
| 39 | DTARI433 | Gene infiltration group | G3 |
| 40 | DTARI746 | Ethiopian native group | G1 |
| 41 | DTARI739 | Bourbon/Typica group | G3 |
| 42 | DTARI743 | Ethiopian native group | G1 |
| 43 | DTARI456 | Ethiopian native group | G3 |
| 44 | DTARI832-1 | Gene infiltration group | G2 |
| 45 | DTARI221 | Bourbon/Typica group | G3 |
| 46 | DTARI409×NB1 | Gene infiltration group | G3 |
| 47 | DTARI519 | Gene infiltration group | G3 |
| 48 | DTARI523 | Gene infiltration group | G2 |
| 49 | DTARI531 | Bourbon/Typica group | G3 |
| 50 | CIFC7963 | Gene infiltration group | G3 |
| 51 | DTARI5 | Bourbon/Typica group | G3 |
| 52 | DTARI6 | Bourbon/Typica group | G3 |
| 53 | DTARI8 | Bourbon/Typica group | G3 |
| 54 | DTARI10 | Bourbon/Typica group | G3 |
| 55 | DTARI12 | Gene infiltration group | G3 |
| 56 | DTARI18 | Gene infiltration group | G2 |
| 57 | DTARI034-2 | Gene infiltration group | G1 |
| 58 | DTARI26 | Gene infiltration group | G3 |
| 59 | DTARI35 | Gene infiltration group | G2 |
| 60 | DTARI47 | Gene infiltration group | G2 |
| 61 | DTARI48 | Gene infiltration group | G2 |
| 62 | DTARI51 | Bourbon/Typica group | G3 |
| 63 | DTARI52 | Bourbon/Typica group | G2 |
| 64 | DTARI55 | Unknown | G3 |
| 65 | DTARI59 | Unknown | G3 |
| 66 | DTARI68 | Bourbon/Typica group | G3 |
| 67 | Typica | Bourbon/Typica group | G3 |
| 68 | DTARI78 | Gene infiltration group | G2 |
| 69 | DTARI94 | Bourbon/Typica group | G3 |
| 70 | DTARI95 | Bourbon/Typica group | G1 |
| 71 | DTARI97 | Gene infiltration group | G3 |
| 72 | DTARI99 | Gene infiltration group | G3 |
| 73 | DTARI143 | Gene infiltration group | G3 |
| 74 | DTARI151 | Gene infiltration group | G2 |
| 75 | DTARI154 | Gene infiltration group | G2 |
| 76 | DTARI157 | Gene infiltration group | G3 |
| 77 | DTARI205 | Bourbon/Typica group | G3 |
| 78 | DTARI206 | Bourbon/Typica group | G3 |
| 79 | DTARI293 | Bourbon/Typica group | G3 |
| 80 | DTARI295 | Unknown | G2 |
| 81 | DTARI297 | Gene infiltration group | G3 |
| 82 | DTARI310 | Unknown | G3 |
| 83 | DTARI311 | Unknown | G3 |
| 84 | DTARI325 | Unknown | G3 |
| 85 | DTARI334 | Unknown | G2 |
| 86 | DTARI337 | Gene infiltration group | G3 |
| 87 | DTARI360 | Gene infiltration group | G2 |
| 88 | DTARI364 | Gene infiltration group | G3 |
| 89 | DTARI370 | Gene infiltration group | G2 |
| 90 | DTARI372 | Unknown | G2 |
| 91 | DTARI373 | Gene infiltration group | G2 |
| 92 | DTARI385 | Gene infiltration group | G3 |
| 93 | DTARI390 | Gene infiltration group | G2 |
| 94 | DTARI393 | Gene infiltration group | G2 |
| 95 | DTARI397 | Gene infiltration group | G2 |
| 96 | DTARI399 | Gene infiltration group | G2 |
| 97 | DTARI400 | Gene infiltration group | G2 |
| 98 | DTARI402 | Gene infiltration group | G2 |
| 99 | DTARI403 | Gene infiltration group | G2 |
| 100 | DTARI404 | Bourbon/Typica group | G3 |
| 101 | DTARI405 | Bourbon/Typica group | G3 |
| 102 | DTARI406 | Bourbon/Typica group | G3 |
| 103 | DTARI407 | Bourbon/Typica group | G3 |
| 104 | DTARI412 | Bourbon/Typica group | G3 |
| 105 | DTARI413 | Ethiopian native group | G1 |
| 106 | DTARI414 | Gene infiltration group | G3 |
| 107 | DTARI416 | Ethiopian native group | G1 |
| 108 | DTARI418 | Gene infiltration group | G2 |
| 109 | DTARI419 | Gene infiltration group | G1 |
| 110 | DTARI420 | Gene infiltration group | G3 |
| 111 | DTARI424 | Gene infiltration group | G3 |
| 112 | DTARI425 | Unknown | G2 |
| 113 | DTARI426 | Unknown | G2 |
| 114 | DTARI428 | Bourbon/Typica group | G3 |
| 115 | DTARI446 | Unknown | G2 |
| 116 | DTARI450 | Gene infiltration group | G3 |
| 117 | DTARI451 | Gene infiltration group | G2 |
| 118 | DTARI454 | Gene infiltration group | G3 |
| 119 | DTARI456 | Ethiopian native group | G1 |
| 120 | DTARI518 | Ethiopian native group | G1 |
| 121 | DTARI525 | Bourbon/Typica group | G3 |
| 122 | DTARI534 | Gene infiltration group | G2 |
| 123 | DTARI538 | Gene infiltration group | G2 |
| 124 | DTARI557 | Unknown | G2 |
| 125 | DTARI561 | Unknown | G3 |
| 126 | DTARI565 | Unknown | G2 |
| 127 | DTARI567 | Unknown | G2 |
| 128 | DTARI576 | Unknown | G3 |
| 129 | DTARI571 | Unknown | G3 |
| 130 | DTARI572 | Unknown | G3 |
| 131 | DTARI585 | Gene infiltration group | G3 |
| 132 | DTARI600 | Bourbon/Typica group | G3 |
| 133 | DTARI606 | Gene infiltration group | G2 |
| 134 | DTARI625 | Bourbon/Typica group | G3 |
| 135 | DTARI626 | Ethiopian native group | G1 |
| 136 | DTARI627 | Ethiopian native group | G1 |
| 137 | DTARI628 | Ethiopian native group | G1 |
| 138 | DTARI634 | Gene infiltration group | G1 |
| 139 | DTARI636 | Gene infiltration group | G2 |
| 140 | DTARI637 | Gene infiltration group | G2 |
| 141 | DTARI638 | Gene infiltration group | G1 |
| 142 | DTARI640 | Gene infiltration group | G2 |
| 143 | DTARI642 | Gene infiltration group | G2 |
| 144 | DTARI673 | Gene infiltration group | G3 |
| 145 | DTARI691 | Unknown | G3 |
| 146 | DTARI693 | Unknown | G2 |
| 147 | DTARI700 | Bourbon/Typica group | G3 |
| 148 | DTARI712 | Bourbon/Typica group | G2 |
| 149 | DTARI759 | Bourbon/Typica group | G2 |
| 150 | DTARI715 | Ethiopian native group | G1 |
| 151 | DTARI716 | Ethiopian native group | G1 |
| 152 | DTARI718 | Ethiopian native group | G1 |
| 153 | DTARI722 | Ethiopian native group | G2 |
| 154 | DTARI724 | Gene infiltration group | G1 |
| 155 | DTARI725 | Gene infiltration group | G1 |
| 156 | DTARI726 | Gene infiltration group | G1 |
| 157 | DTARI727 | Gene infiltration group | G2 |
| 158 | DTARI728 | Gene infiltration group | G2 |
| 159 | DTARI742 | Gene infiltration group | G2 |
| 160 | DTARI743 | Ethiopian native group | G1 |
| 161 | DTARI744 | Gene infiltration group | G2 |
| 162 | DTARI750 | Bourbon/Typica group | G3 |
| 163 | DTARI770 | Ethiopian native group | G3 |
| 164 | DTARI772 | Ethiopian native group | G1 |
| 165 | DTARI774 | Ethiopian native group | G1 |
| 166 | DTARI776 | Ethiopian native group | G1 |
| 167 | DTARI782 | Ethiopian native group | G3 |
| 201 | Co201 | Bourbon/Typica group | G3 |
| 202 | Co202 | Bourbon/Typica group | G3 |
| 203 | Co203 | Bourbon/Typica group | G3 |
| 204 | Co204 | Unknown | G2 |
| 205 | Co205 | Bourbon/Typica group | G3 |
| 206 | Co206 | Bourbon/Typica group | G3 |
| 207 | Co207 | Bourbon/Typica group | G3 |
| 208 | Co208 | Bourbon/Typica group | G3 |
| 209 | Co209 | Bourbon/Typica group | G3 |
| 210 | Co210 | Bourbon/Typica group | G3 |
| 301 | Co301 | Bourbon/Typica group | G3 |
| 302 | Co302 | Bourbon/Typica group | G3 |
| 303 | Co303 | Bourbon/Typica group | G3 |
| 304 | Co304 | Bourbon/Typica group | G3 |
| 401 | Co401 | Bourbon/Typica group | G3 |
| 402 | Co402 | Unknown | G1 |
| 403 | Co403 | Bourbon/Typica group | G3 |
| 63-1 | DTARI52-1 | Bourbon/Typica group | G2 |
