# Supplementary material for: Population structure and genetic diversity of a coffee germplasm collection in China revealed by RAD-seq
Source: Front Plant Sci. 2025 Sep 4;16:1629553. doi: 10.3389/fpls.2025.1629553 (PMC12443757; doi:10.3389/fpls.2025.1629553)
Supplement: Supplementary file 1 [file DataSheet1.zip › Supplementary Materials/Table S2. Statistical table of sequencing data..docx]

**Table S2. Statistical table of sequencing data**. Note: Sample: indicates the sample number; Reads: Number of clean PE reads measured in each sample; Bases: The number of clean bases measured for each sample; GC: The GC content of each sample; Q20: The percentage of bases with a mass number greater than 20; Q30: The percentage of bases with mass numbers greater than 30.

| **Sample** | **Reads** | **Bases** | **GC(%)** | **Q20(%)** | **Q30(%)** |
| --- | --- | --- | --- | --- | --- |
| 1 | 19,679,390 | 5,306,508,374 | 42.55 | 98.86 | 95.82 |
| 2 | 2,507,722 | 671,565,739 | 42.48 | 98.66 | 95.63 |
| 3 | 2,710,542 | 729,733,573 | 41.76 | 98.65 | 95.79 |
| 4 | 2,862,871 | 779,820,803 | 44.77 | 98.51 | 95.36 |
| 5 | 2,481,367 | 662,999,763 | 42.15 | 98.64 | 95.75 |
| 6 | 6,416,207 | 1,724,275,990 | 41.98 | 98.80 | 95.70 |
| 7 | 19,740,522 | 5,307,569,138 | 42.47 | 98.87 | 95.83 |
| 8 | 6,212,057 | 1,688,315,060 | 42.48 | 98.57 | 95.58 |
| 9 | 2,663,086 | 713,021,889 | 42.45 | 98.69 | 95.93 |
| 10 | 5,821,460 | 1,551,809,294 | 43.00 | 98.65 | 95.76 |
| 11 | 6,895,501 | 1,835,334,741 | 42.36 | 98.71 | 95.98 |
| 12 | 3,084,331 | 828,169,908 | 43.16 | 98.65 | 95.75 |
| 13 | 2,345,798 | 629,712,585 | 42.17 | 98.56 | 95.52 |
| 14 | 2,125,953 | 562,414,259 | 42.51 | 98.71 | 95.82 |
| 15 | 7,508,441 | 1,943,260,597 | 41.87 | 98.93 | 96.04 |
| 16 | 2,379,172 | 636,436,636 | 43.88 | 98.54 | 95.42 |
| 17 | 2,595,633 | 689,193,376 | 42.59 | 98.68 | 95.77 |
| 18 | 5,688,009 | 1,519,790,431 | 43.30 | 98.61 | 95.65 |
| 19 | 6,856,181 | 1,743,769,912 | 41.25 | 98.96 | 96.07 |
| 20 | 6,956,320 | 1,825,792,986 | 42.34 | 98.75 | 95.82 |
| 21 | 4,152,929 | 1,111,922,641 | 43.56 | 98.67 | 95.86 |
| 22 | 16,355,729 | 4,187,887,761 | 42.63 | 98.96 | 96.10 |
| 23 | 2,177,502 | 582,608,578 | 42.29 | 98.60 | 95.67 |
| 24 | 2,753,496 | 723,923,602 | 42.67 | 98.72 | 95.79 |
| 25 | 1,548 | 405,487 | 40.17 | 98.74 | 95.50 |
| 26 | 1,941,910 | 520,388,386 | 42.36 | 98.66 | 95.84 |
| 27 | 3,467,448 | 932,775,991 | 41.72 | 98.59 | 95.65 |
| 28 | 5,529,622 | 1,475,345,377 | 42.43 | 98.56 | 95.53 |
| 29 | 13,130,404 | 3,469,847,179 | 42.84 | 98.89 | 95.93 |
| 30 | 2,070,520 | 556,029,206 | 41.95 | 98.64 | 95.72 |
| 31 | 11,792,597 | 3,118,345,628 | 41.96 | 98.86 | 95.81 |
| 32 | 3,612,202 | 955,775,705 | 42.26 | 98.72 | 95.79 |
| 33 | 3,549,689 | 947,451,310 | 41.78 | 98.65 | 95.79 |
| 34 | 2,128,691 | 568,993,578 | 42.19 | 98.71 | 95.98 |
| 35 | 2,413,646 | 646,150,799 | 41.75 | 98.64 | 95.76 |
| 36 | 17,348,979 | 4,588,329,161 | 42.02 | 98.86 | 95.83 |
| 37 | 4,950,313 | 1,319,453,480 | 41.55 | 98.69 | 95.95 |
| 38 | 14,720,057 | 3,959,643,519 | 42.27 | 98.70 | 95.39 |
| 39 | 3,464,393 | 933,082,484 | 42.05 | 98.59 | 95.64 |
| 40 | 6,997,950 | 1,905,763,912 | 42.72 | 98.53 | 95.46 |
| 41 | 5,719,544 | 1,522,373,304 | 42.04 | 98.63 | 95.73 |
| 42 | 16,141,285 | 4,303,501,092 | 42.31 | 98.77 | 95.62 |
| 43 | 6,268,954 | 1,671,182,267 | 42.27 | 98.81 | 95.68 |
| 44 | 7,929,036 | 2,128,301,370 | 42.03 | 98.66 | 95.81 |
| 45 | 3,197,137 | 852,669,452 | 42.18 | 98.62 | 95.63 |
| 46 | 4,455,796 | 1,188,346,742 | 41.85 | 98.84 | 95.78 |
| 47 | 4,961,670 | 1,323,218,856 | 41.96 | 98.66 | 95.85 |
| 48 | 5,731,152 | 1,530,215,376 | 42.10 | 98.71 | 95.98 |
| 49 | 6,930,720 | 1,888,400,447 | 42.64 | 98.47 | 95.26 |
| 50 | 4,042,477 | 1,073,230,921 | 41.90 | 98.66 | 95.85 |
| 51 | 5,388,857 | 1,274,581,873 | 41.81 | 97.62 | 93.99 |
| 52 | 5,615,308 | 1,305,645,579 | 41.63 | 97.62 | 94.07 |
| 53 | 4,061,418 | 1,075,792,949 | 41.97 | 96.58 | 92.10 |
| 54 | 2,889,501 | 743,570,160 | 43.40 | 95.84 | 90.11 |
| 55 | 5,250,885 | 1,233,152,235 | 41.64 | 97.62 | 93.99 |
| 56 | 5,198,690 | 1,392,034,190 | 42.02 | 96.77 | 92.54 |
| 57 | 5,651,618 | 1,382,514,346 | 41.80 | 97.46 | 93.75 |
| 58 | 4,965,753 | 1,244,518,047 | 41.93 | 97.32 | 93.38 |
| 59 | 4,734,141 | 1,124,847,030 | 41.73 | 97.74 | 94.19 |
| 60 | 4,190,718 | 1,031,420,988 | 41.30 | 97.51 | 93.83 |
| 61 | 5,253,305 | 1,273,183,486 | 41.46 | 97.49 | 93.73 |
| 62 | 5,180,380 | 1,264,796,794 | 41.47 | 97.36 | 93.60 |
| 63 | 5,377,051 | 1,268,936,151 | 41.49 | 97.50 | 93.79 |
| 64 | 4,359,053 | 1,035,081,965 | 41.67 | 97.51 | 93.68 |
| 65 | 5,037,339 | 1,245,806,301 | 41.54 | 97.27 | 93.31 |
| 66 | 5,096,045 | 1,173,325,985 | 41.69 | 97.72 | 94.19 |
| 67 | 4,423,358 | 1,031,003,555 | 41.82 | 97.78 | 94.27 |
| 68 | 5,716,620 | 1,342,486,474 | 41.43 | 97.59 | 93.96 |
| 69 | 2,117,941 | 578,726,848 | 43.00 | 96.09 | 90.80 |
| 70 | 1,137,696 | 317,319,437 | 44.23 | 95.33 | 89.08 |
| 71 | 5,098,334 | 1,304,182,714 | 41.59 | 96.97 | 92.92 |
| 72 | 4,931,456 | 1,193,500,005 | 41.62 | 97.50 | 93.74 |
| 73 | 5,871,227 | 1,434,933,103 | 41.41 | 97.36 | 93.60 |
| 74 | 5,255,190 | 1,276,589,888 | 41.20 | 97.34 | 93.47 |
| 75 | 6,145,926 | 1,478,686,793 | 41.66 | 97.24 | 93.37 |
| 76 | 4,940,981 | 1,166,149,482 | 41.82 | 97.28 | 93.40 |
| 77 | 5,647,441 | 1,369,220,218 | 41.44 | 97.28 | 93.40 |
| 78 | 4,932,098 | 1,164,246,340 | 41.16 | 97.54 | 93.87 |
| 79 | 5,081,813 | 1,175,517,598 | 41.68 | 97.68 | 94.18 |
| 80 | 6,854,249 | 1,609,240,047 | 41.24 | 97.37 | 93.73 |
| 81 | 5,095,667 | 1,202,528,621 | 41.51 | 97.47 | 93.75 |
| 82 | 5,305,583 | 1,252,897,042 | 41.59 | 97.53 | 93.90 |
| 83 | 4,487,557 | 1,118,551,917 | 41.65 | 97.03 | 93.09 |
| 84 | 4,744,475 | 1,110,167,589 | 41.89 | 97.57 | 93.90 |
| 85 | 4,652,582 | 1,146,449,713 | 41.69 | 97.22 | 93.35 |
| 86 | 4,857,235 | 1,092,359,523 | 42.79 | 98.25 | 94.73 |
| 87 | 5,461,164 | 1,244,128,274 | 42.18 | 98.29 | 94.83 |
| 88 | 3,605,827 | 815,321,168 | 42.10 | 98.27 | 94.79 |
| 89 | 3,517,955 | 790,481,899 | 42.07 | 98.18 | 94.64 |
| 90 | 3,606,423 | 799,686,792 | 42.29 | 98.31 | 94.90 |
| 91 | 4,027,978 | 905,101,560 | 42.30 | 98.35 | 94.94 |
| 92 | 3,910,877 | 945,256,851 | 42.11 | 98.17 | 94.52 |
| 93 | 4,859,907 | 1,116,917,478 | 42.33 | 98.21 | 94.58 |
| 94 | 5,097,316 | 1,158,200,035 | 42.12 | 98.32 | 94.89 |
| 95 | 4,532,727 | 1,066,773,531 | 41.98 | 98.18 | 94.57 |
| 96 | 4,930,121 | 1,142,436,508 | 42.13 | 98.20 | 94.62 |
| 97 | 3,577,258 | 839,055,986 | 41.73 | 98.16 | 94.51 |
| 98 | 4,638,755 | 1,065,057,851 | 42.13 | 98.10 | 94.37 |
| 99 | 3,681,908 | 868,898,206 | 41.76 | 98.14 | 94.49 |
| 100 | 3,439,783 | 781,326,151 | 42.11 | 98.19 | 94.59 |
| 101 | 4,196,833 | 944,577,917 | 41.68 | 98.12 | 94.47 |
| 102 | 3,510,584 | 792,225,643 | 41.96 | 98.23 | 94.69 |
| 103 | 4,210,823 | 966,478,697 | 41.79 | 97.75 | 94.08 |
| 104 | 2,857,030 | 647,580,047 | 42.20 | 98.29 | 94.90 |
| 105 | 4,306,300 | 992,785,776 | 42.06 | 98.16 | 94.46 |
| 106 | 3,947,197 | 898,658,657 | 42.27 | 98.26 | 94.74 |
| 107 | 2,619,948 | 676,966,093 | 42.06 | 96.05 | 90.50 |
| 108 | 4,123,499 | 947,551,802 | 42.08 | 98.23 | 94.71 |
| 109 | 4,135,184 | 945,740,170 | 41.87 | 98.15 | 94.52 |
| 110 | 5,807,251 | 1,335,000,763 | 41.76 | 98.25 | 94.71 |
| 111 | 3,337,930 | 762,502,852 | 42.54 | 98.23 | 94.64 |
| 112 | 3,069,293 | 744,378,728 | 42.06 | 98.16 | 94.47 |
| 113 | 4,378,595 | 973,933,647 | 42.76 | 98.19 | 94.63 |
| 114 | 3,459,110 | 798,619,782 | 42.36 | 98.12 | 94.40 |
| 115 | 4,518,888 | 1,010,315,028 | 43.25 | 98.34 | 94.94 |
| 116 | 3,747,694 | 889,608,169 | 43.30 | 98.13 | 94.42 |
| 117 | 5,005,488 | 1,128,766,398 | 43.25 | 98.18 | 94.54 |
| 118 | 2,115,480 | 562,144,833 | 43.14 | 97.08 | 92.86 |
| 119 | 3,880,565 | 938,652,653 | 41.82 | 97.00 | 92.51 |
| 120 | 4,030,894 | 939,449,657 | 42.77 | 98.15 | 94.47 |
| 121 | 3,197,649 | 704,409,421 | 42.61 | 98.14 | 94.55 |
| 122 | 3,242,181 | 728,191,630 | 42.68 | 98.18 | 94.54 |
| 123 | 2,511,310 | 691,272,986 | 42.92 | 95.24 | 88.97 |
| 124 | 2,712,052 | 608,493,434 | 42.79 | 98.22 | 94.61 |
| 125 | 3,724,087 | 881,599,916 | 42.97 | 97.38 | 93.38 |
| 126 | 2,755,332 | 637,418,302 | 43.48 | 98.25 | 94.71 |
| 127 | 3,543,592 | 829,531,429 | 42.87 | 98.15 | 94.48 |
| 128 | 4,727,473 | 1,150,756,173 | 42.29 | 97.47 | 93.58 |
| 129 | 2,719,964 | 653,897,626 | 43.46 | 98.09 | 94.35 |
| 130 | 2,600,987 | 664,349,979 | 41.93 | 98.12 | 94.37 |
| 131 | 4,809,685 | 1,172,126,149 | 41.36 | 97.11 | 92.78 |
| 132 | 1,202,579 | 333,561,326 | 43.50 | 94.23 | 87.38 |
| 133 | 3,424,218 | 780,029,860 | 42.19 | 98.14 | 94.55 |
| 134 | 3,324,079 | 897,332,330 | 41.98 | 97.14 | 92.84 |
| 135 | 4,061,246 | 930,799,910 | 42.27 | 98.21 | 94.65 |
| 136 | 4,381,485 | 1,001,413,663 | 42.51 | 98.26 | 94.73 |
| 137 | 3,797,363 | 979,937,560 | 41.80 | 97.20 | 93.04 |
| 138 | 4,343,730 | 980,757,280 | 42.12 | 98.28 | 94.81 |
| 139 | 4,738,352 | 1,094,104,995 | 42.24 | 98.24 | 94.72 |
| 140 | 4,730,222 | 1,103,831,822 | 42.27 | 98.23 | 94.66 |
| 141 | 2,504,774 | 679,400,675 | 41.71 | 96.94 | 92.56 |
| 142 | 4,186,650 | 1,027,164,086 | 42.67 | 98.21 | 94.58 |
| 143 | 4,789,639 | 1,153,248,205 | 41.83 | 97.07 | 92.68 |
| 144 | 3,564,513 | 854,880,708 | 41.49 | 98.11 | 94.42 |
| 145 | 3,840,443 | 872,891,530 | 42.38 | 98.19 | 94.66 |
| 146 | 3,024,592 | 704,827,394 | 43.36 | 98.30 | 94.84 |
| 147 | 3,536,846 | 811,332,604 | 42.42 | 98.34 | 94.96 |
| 148 | 3,281,372 | 760,854,622 | 42.06 | 98.21 | 94.63 |
| 149 | 2,896,453 | 785,707,243 | 41.93 | 97.12 | 92.89 |
| 150 | 3,587,599 | 831,952,859 | 42.07 | 98.29 | 94.77 |
| 151 | 3,042,881 | 694,392,061 | 41.99 | 98.26 | 94.75 |
| 152 | 3,849,428 | 870,769,326 | 43.13 | 98.29 | 94.80 |
| 153 | 3,468,194 | 807,183,495 | 42.77 | 98.25 | 94.69 |
| 154 | 4,912,137 | 1,147,726,445 | 42.20 | 98.22 | 94.60 |
| 155 | 3,460,836 | 866,886,525 | 42.90 | 98.06 | 94.24 |
| 156 | 3,212,063 | 768,659,250 | 42.17 | 98.17 | 94.46 |
| 157 | 3,538,074 | 842,157,311 | 41.98 | 98.23 | 94.64 |
| 158 | 4,488,571 | 1,112,950,057 | 41.50 | 97.36 | 93.57 |
| 159 | 4,998,082 | 1,195,895,135 | 41.76 | 97.67 | 94.09 |
| 160 | 4,989,207 | 1,187,520,354 | 41.60 | 97.60 | 93.92 |
| 161 | 4,610,767 | 1,091,662,387 | 42.45 | 97.62 | 93.92 |
| 162 | 5,362,992 | 1,266,006,055 | 41.31 | 97.62 | 94.05 |
| 163 | 5,047,750 | 1,184,025,119 | 41.87 | 97.58 | 93.94 |
| 164 | 5,110,119 | 1,218,456,735 | 42.17 | 97.49 | 93.73 |
| 165 | 5,820,186 | 1,392,605,829 | 42.08 | 97.44 | 93.74 |
| 166 | 4,184,067 | 1,035,116,289 | 42.29 | 97.46 | 93.53 |
| 167 | 4,069,930 | 970,800,710 | 41.62 | 97.72 | 94.07 |
| 201 | 4,291,417 | 1,045,505,684 | 41.60 | 97.32 | 93.41 |
| 202 | 4,349,245 | 1,091,675,815 | 41.88 | 97.23 | 93.25 |
| 203 | 5,514,828 | 1,391,117,547 | 41.70 | 97.07 | 93.09 |
| 204 | 5,395,393 | 1,372,182,055 | 41.73 | 97.14 | 93.13 |
| 205 | 6,524,521 | 1,620,013,170 | 41.57 | 97.21 | 93.26 |
| 206 | 5,070,081 | 1,236,062,682 | 41.16 | 97.21 | 93.32 |
| 207 | 5,304,501 | 1,293,626,517 | 41.48 | 97.43 | 93.61 |
| 208 | 4,419,631 | 1,038,745,308 | 41.47 | 97.59 | 93.88 |
| 209 | 7,781,690 | 1,835,653,668 | 41.69 | 97.36 | 93.63 |
| 210 | 3,728,056 | 881,737,890 | 41.56 | 97.53 | 93.86 |
| 301 | 1,829,288 | 503,451,653 | 42.70 | 96.24 | 91.17 |
| 302 | 2,220,447 | 549,213,884 | 41.44 | 96.34 | 91.15 |
| 303 | 1,933,376 | 537,042,154 | 42.65 | 95.91 | 90.48 |
| 304 | 3,035,832 | 797,086,498 | 41.96 | 97.14 | 93.02 |
| 401 | 1,984,896 | 557,461,197 | 43.32 | 95.21 | 88.95 |
| 402 | 2,770,424 | 736,420,001 | 43.58 | 95.80 | 90.03 |
| 403 | 1,643,655 | 433,004,456 | 42.13 | 96.78 | 91.97 |
| 63-1 | 4,657,110 | 1,095,947,191 | 41.51 | 97.66 | 94.05 |
| Mean | 4,700,228 | 1,168,532,105 | 42.16 | 97.89 | 94.25 |
| Sum | 869,542,177 | 216,178,439,423 | - | - | - |
