# Supplementary material for: Population structure and genetic diversity of a coffee germplasm collection in China revealed by RAD-seq
Source: Front Plant Sci. 2025 Sep 4;16:1629553. doi: 10.3389/fpls.2025.1629553 (PMC12443757; doi:10.3389/fpls.2025.1629553)
Supplement: Supplementary file 1 [file DataSheet1.zip › Supplementary Materials/Table S3. Statistical table of comparison rates..docx]

**Table S3. Statistical table of comparison rates.** Note: Sample: indicates the sample number; Total reads: The number of reads measured in each sample; Mapped: reads mapped to the genome; Unmapped: reads that cannot be mapped to the genome.

| **Sample** | **Total reads** | **Mapped** | **Mapped%** | **Unmapped** | **Unmapped%** |
| --- | --- | --- | --- | --- | --- |
| 1 | 19,679,390 | 14,964,201 | 76.04% | 4,715,189 | 23.96% |
| 2 | 2,507,722 | 1,998,965 | 79.71% | 508,757 | 20.29% |
| 3 | 2,710,542 | 2,011,829 | 74.22% | 698,713 | 25.78% |
| 4 | 2,862,871 | 1,920,650 | 67.09% | 942,221 | 32.91% |
| 5 | 2,481,367 | 1,844,243 | 74.32% | 637,124 | 25.68% |
| 6 | 6,416,207 | 5,637,190 | 87.86% | 779,017 | 12.14% |
| 7 | 19,740,522 | 16,886,402 | 85.54% | 2,854,120 | 14.46% |
| 8 | 6,212,057 | 4,807,575 | 77.39% | 1,404,482 | 22.61% |
| 9 | 2,663,086 | 2,024,083 | 76.01% | 639,003 | 23.99% |
| 10 | 5,821,460 | 4,487,434 | 77.08% | 1,334,026 | 22.92% |
| 11 | 6,895,501 | 5,563,674 | 80.69% | 1,331,827 | 19.31% |
| 12 | 3,084,331 | 2,253,753 | 73.07% | 830,578 | 26.93% |
| 13 | 2,345,798 | 1,771,207 | 75.51% | 574,591 | 24.49% |
| 14 | 2,125,953 | 1,729,285 | 81.34% | 396,668 | 18.66% |
| 15 | 7,508,441 | 6,769,574 | 90.16% | 738,867 | 9.84% |
| 16 | 2,379,172 | 1,762,890 | 74.10% | 616,282 | 25.90% |
| 17 | 2,595,633 | 2,135,441 | 82.27% | 460,192 | 17.73% |
| 18 | 5,688,009 | 4,444,044 | 78.13% | 1,243,965 | 21.87% |
| 19 | 6,856,181 | 6,230,345 | 90.87% | 625,836 | 9.13% |
| 20 | 6,956,320 | 5,736,592 | 82.47% | 1,219,728 | 17.53% |
| 21 | 4,152,929 | 3,118,480 | 75.09% | 1,034,449 | 24.91% |
| 22 | 16,355,729 | 14,322,438 | 87.57% | 2,033,291 | 12.43% |
| 23 | 2,177,502 | 1,650,301 | 75.79% | 527,201 | 24.21% |
| 24 | 2,753,496 | 2,248,258 | 81.65% | 505,238 | 18.35% |
| 25 | 1,548 | 1,430 | 92.38% | 118 | 7.62% |
| 26 | 1,941,910 | 1,460,164 | 75.19% | 481,746 | 24.81% |
| 27 | 3,467,448 | 2,640,485 | 76.15% | 826,963 | 23.85% |
| 28 | 5,529,622 | 4,510,259 | 81.57% | 1,019,363 | 18.43% |
| 29 | 13,130,404 | 11,458,932 | 87.27% | 1,671,472 | 12.73% |
| 30 | 2,070,520 | 1,542,299 | 74.49% | 528,221 | 25.51% |
| 31 | 11,792,597 | 10,027,114 | 85.03% | 1,765,483 | 14.97% |
| 32 | 3,612,202 | 3,080,617 | 85.28% | 531,585 | 14.72% |
| 33 | 3,549,689 | 2,715,069 | 76.49% | 834,620 | 23.51% |
| 34 | 2,128,691 | 1,620,428 | 76.12% | 508,263 | 23.88% |
| 35 | 2,413,646 | 1,813,642 | 75.14% | 600,004 | 24.86% |
| 36 | 17,348,979 | 14,927,200 | 86.04% | 2,421,779 | 13.96% |
| 37 | 4,950,313 | 3,930,666 | 79.40% | 1,019,647 | 20.60% |
| 38 | 14,720,057 | 11,545,466 | 78.43% | 3,174,591 | 21.57% |
| 39 | 3,464,393 | 2,728,822 | 78.77% | 735,571 | 21.23% |
| 40 | 6,997,950 | 5,242,317 | 74.91% | 1,755,633 | 25.09% |
| 41 | 5,719,544 | 4,639,510 | 81.12% | 1,080,034 | 18.88% |
| 42 | 16,141,285 | 14,654,646 | 90.79% | 1,486,639 | 9.21% |
| 43 | 6,268,954 | 5,590,882 | 89.18% | 678,072 | 10.82% |
| 44 | 7,929,036 | 5,869,553 | 74.03% | 2,059,483 | 25.97% |
| 45 | 3,197,137 | 2,692,081 | 84.20% | 505,056 | 15.80% |
| 46 | 4,455,796 | 3,816,994 | 85.66% | 638,802 | 14.34% |
| 47 | 4,961,670 | 3,823,303 | 77.06% | 1,138,367 | 22.94% |
| 48 | 5,731,152 | 4,727,307 | 82.48% | 1,003,845 | 17.52% |
| 49 | 6,930,720 | 5,290,300 | 76.33% | 1,640,420 | 23.67% |
| 50 | 4,042,477 | 3,358,715 | 83.09% | 683,762 | 16.91% |
| 51 | 5,388,857 | 5,022,550 | 93.20% | 366,307 | 6.80% |
| 52 | 5,615,308 | 5,234,339 | 93.22% | 380,969 | 6.78% |
| 53 | 4,061,418 | 3,802,225 | 93.62% | 259,193 | 6.38% |
| 54 | 2,889,501 | 2,644,930 | 91.54% | 244,571 | 8.46% |
| 55 | 5,250,885 | 4,749,243 | 90.45% | 501,642 | 9.55% |
| 56 | 5,198,690 | 4,530,029 | 87.14% | 668,661 | 12.86% |
| 57 | 5,651,618 | 5,175,722 | 91.58% | 475,896 | 8.42% |
| 58 | 4,965,753 | 4,487,277 | 90.36% | 478,476 | 9.64% |
| 59 | 4,734,141 | 4,370,340 | 92.32% | 363,801 | 7.68% |
| 60 | 4,190,718 | 3,793,177 | 90.51% | 397,541 | 9.49% |
| 61 | 5,253,305 | 4,737,870 | 90.19% | 515,435 | 9.81% |
| 62 | 5,180,380 | 4,755,276 | 91.79% | 425,104 | 8.21% |
| 63 | 5,377,051 | 4,970,618 | 92.44% | 406,433 | 7.56% |
| 64 | 4,359,053 | 4,068,455 | 93.33% | 290,598 | 6.67% |
| 65 | 5,037,339 | 4,656,071 | 92.43% | 381,268 | 7.57% |
| 66 | 5,096,045 | 4,799,338 | 94.18% | 296,707 | 5.82% |
| 67 | 4,423,358 | 4,125,759 | 93.27% | 297,599 | 6.73% |
| 68 | 5,716,620 | 5,302,696 | 92.76% | 413,924 | 7.24% |
| 69 | 2,117,941 | 1,928,138 | 91.04% | 189,803 | 8.96% |
| 70 | 1,137,696 | 1,011,072 | 88.87% | 126,624 | 11.13% |
| 71 | 5,098,334 | 4,691,106 | 92.01% | 407,228 | 7.99% |
| 72 | 4,931,456 | 4,461,606 | 90.47% | 469,850 | 9.53% |
| 73 | 5,871,227 | 5,211,806 | 88.77% | 659,421 | 11.23% |
| 74 | 5,255,190 | 4,736,161 | 90.12% | 519,029 | 9.88% |
| 75 | 6,145,926 | 5,557,225 | 90.42% | 588,701 | 9.58% |
| 76 | 4,940,981 | 4,595,333 | 93.00% | 345,648 | 7.00% |
| 77 | 5,647,441 | 5,079,034 | 89.94% | 568,407 | 10.06% |
| 78 | 4,932,098 | 4,526,276 | 91.77% | 405,822 | 8.23% |
| 79 | 5,081,813 | 4,580,571 | 90.14% | 501,242 | 9.86% |
| 80 | 6,854,249 | 6,339,558 | 92.49% | 514,691 | 7.51% |
| 81 | 5,095,667 | 4,698,096 | 92.20% | 397,571 | 7.80% |
| 82 | 5,305,583 | 4,886,659 | 92.10% | 418,924 | 7.90% |
| 83 | 4,487,557 | 4,152,592 | 92.54% | 334,965 | 7.46% |
| 84 | 4,744,475 | 4,359,688 | 91.89% | 384,787 | 8.11% |
| 85 | 4,652,582 | 4,364,881 | 93.82% | 287,701 | 6.18% |
| 86 | 4,857,235 | 4,638,721 | 95.50% | 218,514 | 4.50% |
| 87 | 5,461,164 | 5,090,554 | 93.21% | 370,610 | 6.79% |
| 88 | 3,605,827 | 3,339,650 | 92.62% | 266,177 | 7.38% |
| 89 | 3,517,955 | 3,340,671 | 94.96% | 177,284 | 5.04% |
| 90 | 3,606,423 | 3,386,907 | 93.91% | 219,516 | 6.09% |
| 91 | 4,027,978 | 3,811,241 | 94.62% | 216,737 | 5.38% |
| 92 | 3,910,877 | 3,661,691 | 93.63% | 249,186 | 6.37% |
| 93 | 4,859,907 | 4,512,432 | 92.85% | 347,475 | 7.15% |
| 94 | 5,097,316 | 4,667,327 | 91.56% | 429,989 | 8.44% |
| 95 | 4,532,727 | 4,187,959 | 92.39% | 344,768 | 7.61% |
| 96 | 4,930,121 | 4,468,649 | 90.64% | 461,472 | 9.36% |
| 97 | 3,577,258 | 3,293,829 | 92.08% | 283,429 | 7.92% |
| 98 | 4,638,755 | 4,248,648 | 91.59% | 390,107 | 8.41% |
| 99 | 3,681,908 | 3,329,623 | 90.43% | 352,285 | 9.57% |
| 100 | 3,439,783 | 3,207,237 | 93.24% | 232,546 | 6.76% |
| 101 | 4,196,833 | 3,939,571 | 93.87% | 257,262 | 6.13% |
| 102 | 3,510,584 | 3,303,956 | 94.11% | 206,628 | 5.89% |
| 103 | 4,210,823 | 3,886,402 | 92.30% | 324,421 | 7.70% |
| 104 | 2,857,030 | 2,693,017 | 94.26% | 164,013 | 5.74% |
| 105 | 4,306,300 | 3,948,991 | 91.70% | 357,309 | 8.30% |
| 106 | 3,947,197 | 3,575,381 | 90.58% | 371,816 | 9.42% |
| 107 | 2,619,948 | 2,356,993 | 89.96% | 262,955 | 10.04% |
| 108 | 4,123,499 | 3,760,992 | 91.21% | 362,507 | 8.79% |
| 109 | 4,135,184 | 3,644,202 | 88.13% | 490,982 | 11.87% |
| 110 | 5,807,251 | 5,174,279 | 89.10% | 632,972 | 10.90% |
| 111 | 3,337,930 | 3,123,379 | 93.57% | 214,551 | 6.43% |
| 112 | 3,069,293 | 2,805,252 | 91.40% | 264,041 | 8.60% |
| 113 | 4,378,595 | 4,100,284 | 93.64% | 278,311 | 6.36% |
| 114 | 3,459,110 | 3,143,351 | 90.87% | 315,759 | 9.13% |
| 115 | 4,518,888 | 4,150,567 | 91.85% | 368,321 | 8.15% |
| 116 | 3,747,694 | 3,397,328 | 90.65% | 350,366 | 9.35% |
| 117 | 5,005,488 | 4,566,612 | 91.23% | 438,876 | 8.77% |
| 118 | 2,115,480 | 1,903,710 | 89.99% | 211,770 | 10.01% |
| 119 | 3,880,565 | 3,547,524 | 91.42% | 333,041 | 8.58% |
| 120 | 4,030,894 | 3,709,704 | 92.03% | 321,190 | 7.97% |
| 121 | 3,197,649 | 2,999,457 | 93.80% | 198,192 | 6.20% |
| 122 | 3,242,181 | 3,030,188 | 93.46% | 211,993 | 6.54% |
| 123 | 2,511,310 | 2,209,201 | 87.97% | 302,109 | 12.03% |
| 124 | 2,712,052 | 2,521,245 | 92.96% | 190,807 | 7.04% |
| 125 | 3,724,087 | 3,403,345 | 91.39% | 320,742 | 8.61% |
| 126 | 2,755,332 | 2,543,705 | 92.32% | 211,627 | 7.68% |
| 127 | 3,543,592 | 3,198,691 | 90.27% | 344,901 | 9.73% |
| 128 | 4,727,473 | 4,118,634 | 87.12% | 608,839 | 12.88% |
| 129 | 2,719,964 | 2,464,054 | 90.59% | 255,910 | 9.41% |
| 130 | 2,600,987 | 2,238,665 | 86.07% | 362,322 | 13.93% |
| 131 | 4,809,685 | 4,456,137 | 92.65% | 353,548 | 7.35% |
| 132 | 1,202,579 | 1,105,044 | 91.89% | 97,535 | 8.11% |
| 133 | 3,424,218 | 3,235,214 | 94.48% | 189,004 | 5.52% |
| 134 | 3,324,079 | 2,940,213 | 88.45% | 383,866 | 11.55% |
| 135 | 4,061,246 | 3,813,760 | 93.91% | 247,486 | 6.09% |
| 136 | 4,381,485 | 4,059,944 | 92.66% | 321,541 | 7.34% |
| 137 | 3,797,363 | 3,566,302 | 93.92% | 231,061 | 6.08% |
| 138 | 4,343,730 | 4,104,782 | 94.50% | 238,948 | 5.50% |
| 139 | 4,738,352 | 4,425,225 | 93.39% | 313,127 | 6.61% |
| 140 | 4,730,222 | 4,364,063 | 92.26% | 366,159 | 7.74% |
| 141 | 2,504,774 | 2,289,932 | 91.42% | 214,842 | 8.58% |
| 142 | 4,186,650 | 3,786,141 | 90.43% | 400,509 | 9.57% |
| 143 | 4,789,639 | 4,499,126 | 93.93% | 290,513 | 6.07% |
| 144 | 3,564,513 | 3,232,743 | 90.69% | 331,770 | 9.31% |
| 145 | 3,840,443 | 3,622,169 | 94.32% | 218,274 | 5.68% |
| 146 | 3,024,592 | 2,830,165 | 93.57% | 194,427 | 6.43% |
| 147 | 3,536,846 | 3,367,259 | 95.21% | 169,587 | 4.79% |
| 148 | 3,281,372 | 2,908,630 | 88.64% | 372,742 | 11.36% |
| 149 | 2,896,453 | 2,572,997 | 88.83% | 323,456 | 11.17% |
| 150 | 3,587,599 | 3,321,637 | 92.59% | 265,962 | 7.41% |
| 151 | 3,042,881 | 2,796,474 | 91.90% | 246,407 | 8.10% |
| 152 | 3,849,428 | 3,634,505 | 94.42% | 214,923 | 5.58% |
| 153 | 3,468,194 | 3,207,058 | 92.47% | 261,136 | 7.53% |
| 154 | 4,912,137 | 4,587,311 | 93.39% | 324,826 | 6.61% |
| 155 | 3,460,836 | 3,185,161 | 92.03% | 275,675 | 7.97% |
| 156 | 3,212,063 | 2,945,037 | 91.69% | 267,026 | 8.31% |
| 157 | 3,538,074 | 3,266,651 | 92.33% | 271,423 | 7.67% |
| 158 | 4,488,571 | 4,085,823 | 91.03% | 402,748 | 8.97% |
| 159 | 4,998,082 | 4,589,270 | 91.82% | 408,812 | 8.18% |
| 160 | 4,989,207 | 4,621,289 | 92.63% | 367,918 | 7.37% |
| 161 | 4,610,767 | 4,207,804 | 91.26% | 402,963 | 8.74% |
| 162 | 5,362,992 | 5,026,834 | 93.73% | 336,158 | 6.27% |
| 163 | 5,047,750 | 4,555,112 | 90.24% | 492,638 | 9.76% |
| 164 | 5,110,119 | 4,753,034 | 93.01% | 357,085 | 6.99% |
| 165 | 5,820,186 | 5,414,266 | 93.03% | 405,920 | 6.97% |
| 166 | 4,184,067 | 3,796,688 | 90.74% | 387,379 | 9.26% |
| 167 | 4,069,930 | 3,821,778 | 93.90% | 248,152 | 6.10% |
| 201 | 4,291,417 | 3,954,395 | 92.15% | 337,022 | 7.85% |
| 202 | 4,349,245 | 3,956,007 | 90.96% | 393,238 | 9.04% |
| 203 | 5,514,828 | 5,114,504 | 92.74% | 400,324 | 7.26% |
| 204 | 5,395,393 | 5,028,550 | 93.20% | 366,843 | 6.80% |
| 205 | 6,524,521 | 5,986,396 | 91.75% | 538,125 | 8.25% |
| 206 | 5,070,081 | 4,732,646 | 93.34% | 337,435 | 6.66% |
| 207 | 5,304,501 | 5,053,996 | 95.28% | 250,505 | 4.72% |
| 208 | 4,419,631 | 4,145,052 | 93.79% | 274,579 | 6.21% |
| 209 | 7,781,690 | 7,341,974 | 94.35% | 439,716 | 5.65% |
| 210 | 3,728,056 | 3,571,436 | 95.80% | 156,620 | 4.20% |
| 301 | 1,829,288 | 1,658,956 | 90.69% | 170,332 | 9.31% |
| 302 | 2,220,447 | 2,061,237 | 92.83% | 159,210 | 7.17% |
| 303 | 1,933,376 | 1,762,283 | 91.15% | 171,093 | 8.85% |
| 304 | 3,035,832 | 2,673,267 | 88.06% | 362,565 | 11.94% |
| 401 | 1,984,896 | 1,700,336 | 85.66% | 284,560 | 14.34% |
| 402 | 2,770,424 | 2,535,947 | 91.54% | 234,477 | 8.46% |
| 403 | 1,643,655 | 1,500,805 | 91.31% | 142,850 | 8.69% |
| 63-1 | 4,657,110 | 4,358,543 | 93.59% | 298,567 | 6.41% |
| Mean | 4,700,228 | 4,157,884 | 88.77% | 542,344 | 11.23% |
