# Supplementary material for: Population structure and genetic diversity of a coffee germplasm collection in China revealed by RAD-seq
Source: Front Plant Sci. 2025 Sep 4;16:1629553. doi: 10.3389/fpls.2025.1629553 (PMC12443757; doi:10.3389/fpls.2025.1629553)
Supplement: Supplementary file 1 [file DataSheet1.zip › Supplementary Materials/Table S4. Statistical table of sequencing coverage and coverage depth..docx]

**Table S4. Statistical table of sequencing coverage and coverage depth.** Note: Sample: indicates the sample number; Coverage: The percentage of sequencing data that covers the entire genome; Mean Depth: average coverage depth.

| **Sample** | **Coverage (%)** | **Mean depth** |
| --- | --- | --- |
| 1 | 4.88 | 40.03 |
| 2 | 3.27 | 8.99 |
| 3 | 3.50 | 8.60 |
| 4 | 2.79 | 10.25 |
| 5 | 3.25 | 8.21 |
| 6 | 3.76 | 22.50 |
| 7 | 6.08 | 39.00 |
| 8 | 4.16 | 16.48 |
| 9 | 2.89 | 8.88 |
| 10 | 4.15 | 15.35 |
| 11 | 4.83 | 16.80 |
| 12 | 3.20 | 9.79 |
| 13 | 2.94 | 9.04 |
| 14 | 2.98 | 8.37 |
| 15 | 3.32 | 27.71 |
| 16 | 2.77 | 9.25 |
| 17 | 3.10 | 10.36 |
| 18 | 3.87 | 16.56 |
| 19 | 5.84 | 14.16 |
| 20 | 4.26 | 19.00 |
| 21 | 3.48 | 12.18 |
| 22 | 5.08 | 36.70 |
| 23 | 2.79 | 8.69 |
| 24 | 2.93 | 10.55 |
| 25 | 0.01 | 0.83 |
| 26 | 2.66 | 7.90 |
| 27 | 3.49 | 11.32 |
| 28 | 4.36 | 15.61 |
| 29 | 4.49 | 36.21 |
| 30 | 3.12 | 7.31 |
| 31 | 5.32 | 25.70 |
| 32 | 3.50 | 13.06 |
| 33 | 3.46 | 10.31 |
| 34 | 2.95 | 7.89 |
| 35 | 3.18 | 8.59 |
| 36 | 6.77 | 31.05 |
| 37 | 4.25 | 13.94 |
| 38 | 5.03 | 34.33 |
| 39 | 4.68 | 8.08 |
| 40 | 4.41 | 17.52 |
| 41 | 4.34 | 14.97 |
| 42 | 8.47 | 24.33 |
| 43 | 3.38 | 24.36 |
| 44 | 5.69 | 14.98 |
| 45 | 3.55 | 11.41 |
| 46 | 2.95 | 18.79 |
| 47 | 4.08 | 12.82 |
| 48 | 3.94 | 17.74 |
| 49 | 4.16 | 19.73 |
| 50 | 3.43 | 14.28 |
| 51 | 3.03 | 21.47 |
| 52 | 3.15 | 20.98 |
| 53 | 2.50 | 23.01 |
| 54 | 2.19 | 15.66 |
| 55 | 3.34 | 17.59 |
| 56 | 1.52 | 44.12 |
| 57 | 2.79 | 24.17 |
| 58 | 2.78 | 22.81 |
| 59 | 2.55 | 21.85 |
| 60 | 2.89 | 17.28 |
| 61 | 2.96 | 20.00 |
| 62 | 2.86 | 23.07 |
| 63 | 3.08 | 20.62 |
| 64 | 2.69 | 18.87 |
| 65 | 2.67 | 24.53 |
| 66 | 3.08 | 18.48 |
| 67 | 2.99 | 17.77 |
| 68 | 2.77 | 24.77 |
| 69 | 2.65 | 10.98 |
| 70 | 1.61 | 9.02 |
| 71 | 2.89 | 24.44 |
| 72 | 2.72 | 22.15 |
| 73 | 3.19 | 22.19 |
| 74 | 3.00 | 20.52 |
| 75 | 3.25 | 21.66 |
| 76 | 2.92 | 20.26 |
| 77 | 3.12 | 21.31 |
| 78 | 3.05 | 18.75 |
| 79 | 3.06 | 17.87 |
| 80 | 3.27 | 25.08 |
| 81 | 2.96 | 20.27 |
| 82 | 2.67 | 23.60 |
| 83 | 2.77 | 20.96 |
| 84 | 3.10 | 17.41 |
| 85 | 2.52 | 23.75 |
| 86 | 2.68 | 20.33 |
| 87 | 2.55 | 23.66 |
| 88 | 2.34 | 17.45 |
| 89 | 2.50 | 15.91 |
| 90 | 2.64 | 14.56 |
| 91 | 2.63 | 17.36 |
| 92 | 2.32 | 21.01 |
| 93 | 2.35 | 22.74 |
| 94 | 2.59 | 21.13 |
| 95 | 2.86 | 18.63 |
| 96 | 2.71 | 20.01 |
| 97 | 2.38 | 17.08 |
| 98 | 3.01 | 16.80 |
| 99 | 2.55 | 16.04 |
| 100 | 2.75 | 14.55 |
| 101 | 3.05 | 15.81 |
| 102 | 2.73 | 14.64 |
| 103 | 3.96 | 12.24 |
| 104 | 2.62 | 12.59 |
| 105 | 3.05 | 15.72 |
| 106 | 2.66 | 15.73 |
| 107 | 2.12 | 16.21 |
| 108 | 2.79 | 16.64 |
| 109 | 1.26 | 32.78 |
| 110 | 2.99 | 20.80 |
| 111 | 2.82 | 13.45 |
| 112 | 2.23 | 17.42 |
| 113 | 2.66 | 18.08 |
| 114 | 2.88 | 13.43 |
| 115 | 2.78 | 17.76 |
| 116 | 2.89 | 15.02 |
| 117 | 2.87 | 18.94 |
| 118 | 2.11 | 13.96 |
| 119 | 2.79 | 14.93 |
| 120 | 2.89 | 15.75 |
| 121 | 3.08 | 11.18 |
| 122 | 2.51 | 13.94 |
| 123 | 2.30 | 14.56 |
| 124 | 2.50 | 11.59 |
| 125 | 3.01 | 14.77 |
| 126 | 2.45 | 12.65 |
| 127 | 2.81 | 13.51 |
| 128 | 3.53 | 14.93 |
| 129 | 2.40 | 13.35 |
| 130 | 2.80 | 11.53 |
| 131 | 3.11 | 17.99 |
| 132 | 2.09 | 7.89 |
| 133 | 2.51 | 15.56 |
| 134 | 2.56 | 18.40 |
| 135 | 2.51 | 18.42 |
| 136 | 2.65 | 19.02 |
| 137 | 2.57 | 21.08 |
| 138 | 3.09 | 15.74 |
| 139 | 2.96 | 18.04 |
| 140 | 2.96 | 17.95 |
| 141 | 2.44 | 15.31 |
| 142 | 2.26 | 22.25 |
| 143 | 2.97 | 17.67 |
| 144 | 1.98 | 22.11 |
| 145 | 2.59 | 16.70 |
| 146 | 2.47 | 14.06 |
| 147 | 2.68 | 15.53 |
| 148 | 2.75 | 12.35 |
| 149 | 2.88 | 13.88 |
| 150 | 2.88 | 14.06 |
| 151 | 2.75 | 11.89 |
| 152 | 2.71 | 14.85 |
| 153 | 2.49 | 15.27 |
| 154 | 2.63 | 20.22 |
| 155 | 2.83 | 14.97 |
| 156 | 2.79 | 13.45 |
| 157 | 2.55 | 16.02 |
| 158 | 2.66 | 20.66 |
| 159 | 1.53 | 34.53 |
| 160 | 3.34 | 17.67 |
| 161 | 1.69 | 29.02 |
| 162 | 3.27 | 20.22 |
| 163 | 3.37 | 17.21 |
| 164 | 2.66 | 21.85 |
| 165 | 3.12 | 22.27 |
| 166 | 3.19 | 15.95 |
| 167 | 2.98 | 16.84 |
| 201 | 2.99 | 18.02 |
| 202 | 3.17 | 17.81 |
| 203 | 3.21 | 22.30 |
| 204 | 3.08 | 22.86 |
| 205 | 3.03 | 27.39 |
| 206 | 4.58 | 13.79 |
| 207 | 2.85 | 23.99 |
| 208 | 2.80 | 18.91 |
| 209 | 3.00 | 28.89 |
| 210 | 2.74 | 16.88 |
| 301 | 2.30 | 10.75 |
| 302 | 2.12 | 12.99 |
| 303 | 2.43 | 10.66 |
| 304 | 2.51 | 15.96 |
| 401 | 1.88 | 13.90 |
| 402 | 1.95 | 16.56 |
| 403 | 2.13 | 10.22 |
| 63-1 | 2.78 | 19.88 |
| Mean | 3.05 | 17.66 |
